# The role of trimethylamine N-oxide in disease pathogenesis and the therapeutic potential of traditional Chinese medicine

**DOI:** 10.3389/fphar.2025.1592524

**Published:** 2025-07-24

**Authors:** Zizhen Qin, Wanning Wu, Xinyu Yang, Xiao Wang, Anni Ding, Yueyi Huang, Jiaxuan Tang, Siyuan Jiang, Peng Zhang, Chenwei Qian, Xiangrui Zhang, Shihan Zhou, Yanqing Wang, Zhouchenghao Song, Minjie Sun, Mingqiang Wang, Shuang Shen, Boran Zhu

**Affiliations:** ^1^ Nanjing University of Chinese Medicine, Nanjing, China; ^2^ School of Elderly Care Services and Management, Nanjing University of Chinese Medicine, Nanjing, China; ^3^ Union Laboratory of Traditional Chinese Medicine for Brain Science and Gerontology, Nanjing University of Chinese Medicine, Nanjing, China; ^4^ Jiangsu Association of Medicated Diet, Nanjing, China

**Keywords:** trimethylamine N-oxide, gut microbiota, chronic diseases, traditional Chinese medicine, inflammation, oxidative stress, metabolic disorders

## Abstract

Trimethylamine N-oxide (TMAO), a metabolite derived from gut microbiota, has been implicated in the pathogenesis of various chronic diseases, including cardiovascular, digestive, neurological, and renal disorders. This review explores the complex mechanisms by which TMAO contributes to disease progression, including its role in inflammation, oxidative stress, and metabolic disorders. The study focused on the potential of traditional Chinese medicine (TCM) to regulate TMAO levels and mitigate its adverse effects. TCM interventions, through modulation of gut microbiota and inhibition of key enzymes like flavin-containing monooxygenase 3 (FMO3), offer promising therapeutic avenues. Despite the positive outcomes observed in preliminary studies, further research is needed to fully elucidate the mechanisms by which TCM interacts with TMAO and to establish its efficacy in clinical settings.

## Graphical Abstract

**Figure:**
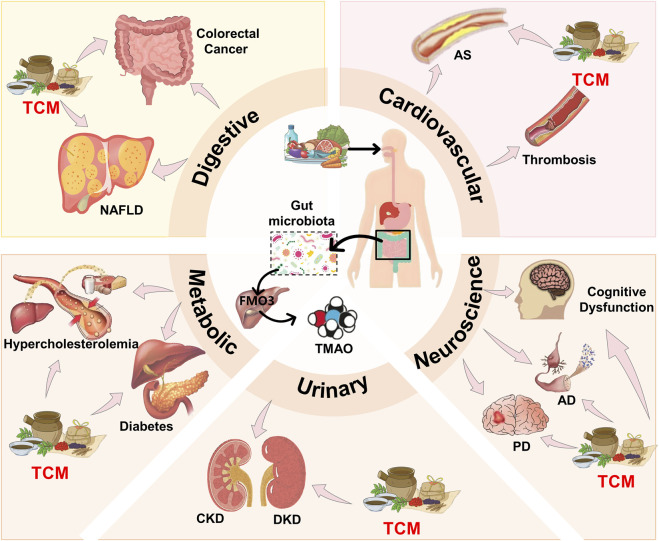
Overview of Gut Microbiota Metabolite TMAO.

## 1 Introduction

Trimethylamine N-oxide (TMAO) is a bioactive molecule derived from metabolites of the gut microbiota, which is converted from trimethylamine (TMA) by the flavin containing monooxygenase 3 (FMO3) in the host liver. Studies have indicated a positive correlation between TMAO levels and various chronic non-communicable diseases, including insulin resistance, atherosclerotic plaque formation, diabetes, cancer, heart failure, hypertension, chronic kidney disease, liver disease, neurodegeneration, and Alzheimer’s disease (Coutinho-Wolino et al., 2021). Dietary choline is associated with increased plasma TMAO concentrations, thereby raising the likelihood of adverse cardiovascular events, metabolic disorders, neurological diseases, and renal diseases (Agus et al., 2021). Traditional Chinese Medicine (TCM) is a holistic medical system that utilizes natural plant or animal-based substances as methods for treating diseases and has a history of over five thousand years. With increased international exchange, TCM has also been adopted in many other countries, such as the United States, Canada, Finland, Australia, the United Kingdom, and others, and encompasses a variety of therapeutic practices. Specifically, TCM includes herbal preparations, acupuncture, moxibustion, dietary therapy (medicinal cuisine), tuina (therapeutic massage), and other traditional approaches. In this review, “TCM” primarily refers to these modalities, with particular emphasis on herbal interventions and dietary therapy, while also acknowledging the important role of practices such as acupuncture in the prevention and treatment of diseases related to TMAO (Wu et al., 2012).

### 1.1 Sources of TMAO

In the human body, TMA is a significant precursor of TMAO. TMA is primarily formed in the gut through the enzymatic metabolism of certain dietary compounds present in foods such as peanuts, dairy products, liver, egg yolks, and other full-fat dietary items, which all characterized by high levels of choline and carnitine, being both important precursors of TMA and TMAO (Wu K. et al., 2020). Choline, a trimethylamine-containing compound, as part of the phosphatidylcholine head group, can be found in various foods. Phosphatidylcholine, also known as lecithin, is a fundamental component of membranes and the neurotransmitter acetylcholine. Phospholipase D can catalyze the conversion of lecithin to choline, which is a reversible transformation (Fennema et al., 2016). Human milk and soy-derived infant formula contain substantial amounts of free choline, while beef liver, cauliflower, and peanuts (Zeisel et al., 2003) contain several choline compounds (phosphatidylcholine, phosphocholine, sphingomyelin, *etc.*). Studies have shown that the key enzyme responsible for producing TMA is choline TMA-lyase (CutC), which is a glycyl radical enzyme requiring the activating protein CutD to assist its function. These two components work together to catalyze the cleavage of choline, generating TMA and acetaldehyde (Yoo et al., 2021). The process of TMA production through the choline TMA-lyase complex, namely, CutC and CutD (often collectively referred to as CUTC), represents the primary pathway by which gut microbiota convert dietary choline into TMA (Craciun and Balskus, 2012).

L-carnitine is present in red meat and dairy products (Feller and Rudman, 1988). *Serratia* bacteria and *Acinetobacter* calcoaceticus that found in the human gut can cleave the 3-hydroxybutyryloxy group of L-carnitine to directly produce TMA (Meadows and Wargo, 2015). Certain gut microbial communities possess two enzyme systems, namely, the CNTA/B systems, which are composed of the Rieske-type oxygenase CntA and the electron transfer reductase CntB. These two subunits collaborate to degrade L-carnitine *in vitro*, thereby generating TMA (Zhu et al., 2014). Moreover, within the body, carnitine can be metabolized into γ-butyrobetaine and crotonobetaine through the enzymatic action of L-carnitine dehydrogenase (Meadows and Wargo, 2015) and γ-butyrobetaine CoA transferase (Koeth et al., 2014), while choline can be oxidized into betaine by choline dehydrogenase and betaine aldehyde dehydrogenase. Betaine itself is a substance abundant in wheat bran, wheat germ, and spinach (Zeisel et al., 2003). These compounds can also serve as precursors for the formation of TMA and TMAO (Wang et al., 2019). Betaine can be reduced and cleaved into TMA and acetate in a coupled redox process (Stickland reaction) by betaine reductase (Naumann et al., 1983). In addition to these common sources, TMA can also be derived from dietary ergothioneine found in foods such as legumes, mushrooms, and liver (Cheah and Halliwell, 2012). Ergothionase catalyzes the degradation of ergothioneine to produce TMA and the by-product thiosulfonate (Muramatsu et al., 2013). Once the above precursors are converted into TMA through complex actions by gut microbiota and enzymes, a small portion of TMA can be directly metabolized into TMAO and dimethylamine (DMA) by bacteria in the gut. The remainder can be transported to the liver *via* the portal vein circulation, and in there it would be oxidized into TMAO by the host liver flavin monooxygenases (FMO1 and FMO3) (Lang et al., 1998; Descamps et al., 2019). Finally, TMAO and TMA can also be directly acquired from fish and other seafood (Tang and Hazen, 2017). Compared to freshwater fish, the concentration of TMA in marine fish (Bain et al., 2005) (such as cod, halibut, herring, and skate) is higher.

### 1.2 Metabolism of TMAO

After being produced in the body, the majority of TMA is absorbed *via* passive diffusion across the intestinal cell membrane. Subsequently, almost 95% of TMA is oxidized to TMAO in the liver. Before excretion, TMAO—and any unmetabolized TMA—enters the plasma and is transported to body tissues (such as the lungs, liver, kidneys, muscles, and heart) where it accumulates as an osmolyte compound. Relatively, TMA and TMAO are most likely to accumulate in the lungs and kidneys, followed by the liver, then muscles, with the heart being the least likely organ for accumulation (Smith et al., 1994). Eventually, both TMA and TMAO are mixed with urine in the kidneys *via* the circulatory system and excreted from the body. Organic cation transporter 2 (OCT2), situated on the basolateral membrane of renal tubular cells, serves as a crucial uptake transporter for TMAO, with over 90% of TMAO being excreted in the urine after renal metabolism (Bennett et al., 2013; Canyelles et al., 2018). Approximately 4% of the remaining TMAO is excreted *via* faeces, and less than 1% is expelled through respiration. Research indicates that most orally ingested TMAO can be absorbed by extrahepatic tissues without microbial or hepatic processing. Under the action of TMAO reductase, some TMAO can be reduced back to TMA in the intestine. Additionally, certain bacteria have the ability to convert TMA and TMAO into DMA and formaldehyde through trimethylamine dehydrogenase (TMADH) and TMAO demethylase.

## 2 Treatment approaches related to TMAO

The above content suggests that the majority of TMA is absorbed into the hepatic portal venous circulation *via* passive diffusion across the intestinal cell membrane and subsequently converted to TMAO by hepatic FMO3, and TMAO elimination can be achieved by targeting specific pathways, including inhibition of TMA precursor production, suppression of TMA production, and blocking the conversion of TMA to TMAO.

### 2.1 Inhibition of TMA and TMAO production

By supplementing with broad-spectrum antibiotics (e.g., ciprofloxacin and metronidazole) (Tang et al., 2013), it is possible to inhibit microbial groups that can convert choline, betaine, and L-carnitine into TMA (Wang et al., 2011). Although antibiotics initially suppress TMAO levels, the long-term persistence of this effect remains unknown. Unlike this mechanism, Meldonium is an anti-ischaemic and anti-atherosclerotic drug that can competitively inhibit not only butyrobetaine hydroxylase but also the reabsorption of L-carnitine in the kidneys *via* the carnitine/organic cation transporter protein (OCTN2). Moreover, it also reduces TMAO concentration in human plasma by increasing urinary excretion (Dambrova et al., 2013). In terms of the microbial metabolic regulation, a structural analogue of choline, 3,3-dimethyl-1-butanol (DMB), does not impede choline uptake into cells. However, it can restrain the formation of TMA by microorganisms through inhibiting the activity of microbial TMA lyases, such as CutC (Yang Y. et al., 2022), and reducing the formation of TMA in various human microbiomes. Irreversible covalent inhibitors targeting CutC lyase also exert analogous effects. Previous studies have demonstrated that fluoromethylcholine (FMC), a non-lethal, microbe-friendly inhibitor of CutC lyase, significantly reduces plasma TMAO levels by irreversibly modifying the enzymatic pair CutC/D within gut microbiota harboring the corresponding gene cluster (Benson et al., 2023). The research by Nilaksh Gupta and colleagues found that iodomethylcholine (IMC) can inhibit the production of microbial TMA in hosts and reduce plasma TMAO levels, demonstrating over 10,000-fold greater potency (sub-nanomolar IC50) compared to previously reported microbial choline TMA lyase inhibitors (Gupta et al., 2020). In addition, the reversible competitive inhibitor betaine aldehyde can lower plasma TMAO levels by specifically targeting the gut microbial enzyme CutC (Orman et al., 2019). DMB treatment significantly lowers plasma TMAO levels and prevents cardiac dysfunction (Wang et al., 2015), without affecting body weight and dyslipidemia (Chen K. et al., 2017). It is worth noting that in addition to the strategies of directly intervening in metabolic pathways mentioned above, Enalapril, an angiotensin-converting enzyme inhibitor (ACE-I), represents a new method of reducing plasma TMAO levels, although the underlying mechanism has not been discovered (Konop et al., 2018). Additionally, recent studies have confirmed that inhibiting host enzyme FMO3 has a positive impact on reducing circulating TMAO levels and diet-enhanced atherosclerosis. However, adverse side effects may be brought by inhibiting FMO3, including liver inflammation and trimethylaminuria (fish odour syndrome) (Wang et al., 2015).

### 2.2 Microbiota-based treatments to reduce TMAO

TMAO represents a critical microbial metabolite derived from the gut microbiota’s metabolism of dietary nutrients. Consequently, reducing TMAO levels *via* microbiota-targeted approaches has emerged as a significant research focus for treating associated diseases. Wang et al. demonstrated that berberine (BBR) reduces gut microbial TMA biosynthesis, ultimately lowering plasma TMAO levels, by decreasing the abundance of CutC/D-expressing bacterial taxa, including Lachnospira, Lachnospiraceae group, Lachnospiraceae group, Clostridia, Lachnoclostridium, and Ruminococcus (Wang et al., 2024). In contrast, puerarin (PU) acts by targeting specific TMA-producing species. It specifically inhibits the membrane function of Prevotella copri, thereby diminishing its capacity to generate TMA and subsequently reducing TMAO levels (Authors, 2024). Furthermore, remodeling the gut microbiota structure can reduce the colonization and metabolic activity of bacteria that produce TMA precursors, leading to decreased TMAO concentrations. Recent research revealed that Akkermansia muciniphila, a mucin-degrading bacterium with probiotic properties, secretes the antimicrobial peptide Amuc. This peptide inhibits the growth of TMA-producing bacteria such as Anaerococcus hydrogenalis, resulting in reduced TMAO levels (Li et al., 2024). The bioactive xanthone mangiferin lowers plasma TMAO by reshaping gut microbial composition; it promotes the growth of beneficial taxa including Akkermansia, Parabacteroides, and Bifidobacteriaceae, while concurrently reducing the relative abundance of the pathobiont genus *Helicobacter* (He Z. et al., 2023). Additionally, Lactiplantibacillus plantarum ZDY04 achieves therapeutic effects by modulating gut microbiota structure, significantly decreasing serum TMAO content and cecal TMA levels (Qiu et al., 2018). Collectively, these findings indicate that microbiota-targeted therapy offers a promising therapeutic paradigm for the precise intervention of TMAO-related diseases.

### 2.3 Negative effects

TMAO is a stable, non-volatile and odourless oxidised product (Wang et al., 2011), whereas TMA is a volatile gas with a fishy odour. Mutations or inhibitions of human FMO3 that prevent TMAO production can lead to the accumulation of TMA, which is excreted in excess in urine, sweat, and breath, smelling like rotten fish (Zeisel and Warrier, 2017). Thus, inhibiting the conversion of TMA to TMAO by reducing FMO3 expression could result in fish odour syndrome. Moreover, while antibiotic treatment can suppress plasma TMAO levels, such as with metronidazole and ciprofloxacin, continued use of antibiotics to lower TMAO concentrations can lead to resistance in bacterial strains. Moreover, antibiotics can kill beneficial bacteria as well as harmful ones, leading to gut dysbiosis, and after stopping antibiotics, TMAO levels rise again. Long-term use of Meldonium may cause side effects such as hypoxia, dizziness, and reduced blood supply (Benedetto et al., 2008). Taking ACE-Is may impair kidney function, potentially resulting in renal failure and electrolyte imbalance, and enalapril treatment may cause increased water intake (Konop et al., 2018). Additionally, the accumulation of TMA and its unpleasant odour in individuals with FMO3 gene defection who suffer from fish odour syndrome diminishes the potential of FMO3 as an inhibitory therapeutic target. Therefore, this paper will analyze the advantages of Traditional Chinese Medicine in treating diseases related to TMAO.

## 3 The role of TMAO in various diseases and the therapeutic effects of traditional Chinese medicine

Multiple studies have shown that TMAO is involved in the occurrence and development of various chronic diseases, including cardiovascular, digestive, neurological, kidney diseases, and metabolic disorders. Modern medical drugs have been widely used for treatment, but there are various side effects. At the same time, TCM has been proven to have significant potential and remarkable effects in improving and treating chronic diseases. Based on its multi-component and multi-target characteristics, TCM offers a unique therapeutic approach for regulating TMAO levels and mitigating the progression of related diseases. In this review, herbal medicine is identified as one of the primary modalities of TCM for treating TMAO-related conditions, demonstrating strong potential in modulating the gut microbiota, inhibiting key enzymes such as FMO3, and reducing systemic inflammation and oxidative stress. For example, berberine, baicalin, and curcumin can lower serum TMAO by altering gut microbial composition or intervening in metabolic pathways (Liu et al., 2020; Wang et al., 2024). Dietary and nutritional interventions can influence TMAO production by promoting a healthier gut microbiota. Studies have shown that dietary fiber, a nutrient digested and absorbed in the colon, can significantly lower TMAO levels in human peripheral blood (Xie et al., 2025). Other modalities, such as acupuncture and moxibustion, are believed to improve organ function and overall regulation by modulating neuroimmune pathways and reducing inflammatory responses, although their specific impact on TMAO requires further investigation (Sun et al., 2024). The combination and individualized use of these TCM approaches provide both theoretical and clinical foundations for the comprehensive prevention and treatment of TMAO-related chronic diseases. The subsequent sections of this review will further explore the therapeutic potential and mechanisms of TCM in addressing TMAO-related conditions.

### 3.1 Digestive system and metabolic diseases

Numerous studies indicate that TMAO is closely associated with digestive system diseases such as colorectal cancer (CRC) and non-alcoholic fatty liver disease (NAFLD). TMAO activates its intracellular potential receptors, protein kinase R-like endoplasmic reticulum kinase (PERK), which further activates NLRP3 and NF-κB that mediate pro-inflammatory responses. TMAO also induces reactive oxygen species through oxidative stress, altering the invasion and migration of tumour cells, thereby affecting CRC progression (Duizer and de Zoete, 2023). Moreover, TMAO can directly enhance the onset of NAFLD through oxidative stress or, due to disorders in hepatic lipid metabolism and inflammation, affect bile acid production, alter liver TG levels, influence cholesterol transport and glucose and energy balance, thus exacerbating hepatic steatosis. Metabolic disorders are an increasingly severe global health issue, closely linked to changes in the intestinal microbiome. TMAO is an important regulator of lipid metabolism and is closely related to the pathogenesis of metabolic diseases such as hypercholesterolemia and diabetes (Agus et al., 2021). TMAO inhibits reverse cholesterol transport (RCT), reduces hepatic bile acid transporters, and alters bile acid synthesis, leading to impaired cholesterol elimination and hypercholesterolemia, thereby increasing cardiovascular disease risk (Koeth et al., 2013). Furthermore, TMAO promotes diabetes by elevating fasting insulin, increasing insulin resistance (HOMA-IR), and inducing adipose tissue inflammation, contributing to glucose metabolism dysfunction and heightened cardiovascular event risks in diabetic patients (Subramaniam and Fletcher, 2018).

#### 3.1.1 Colorectal cancer

**FIGURE 1:**
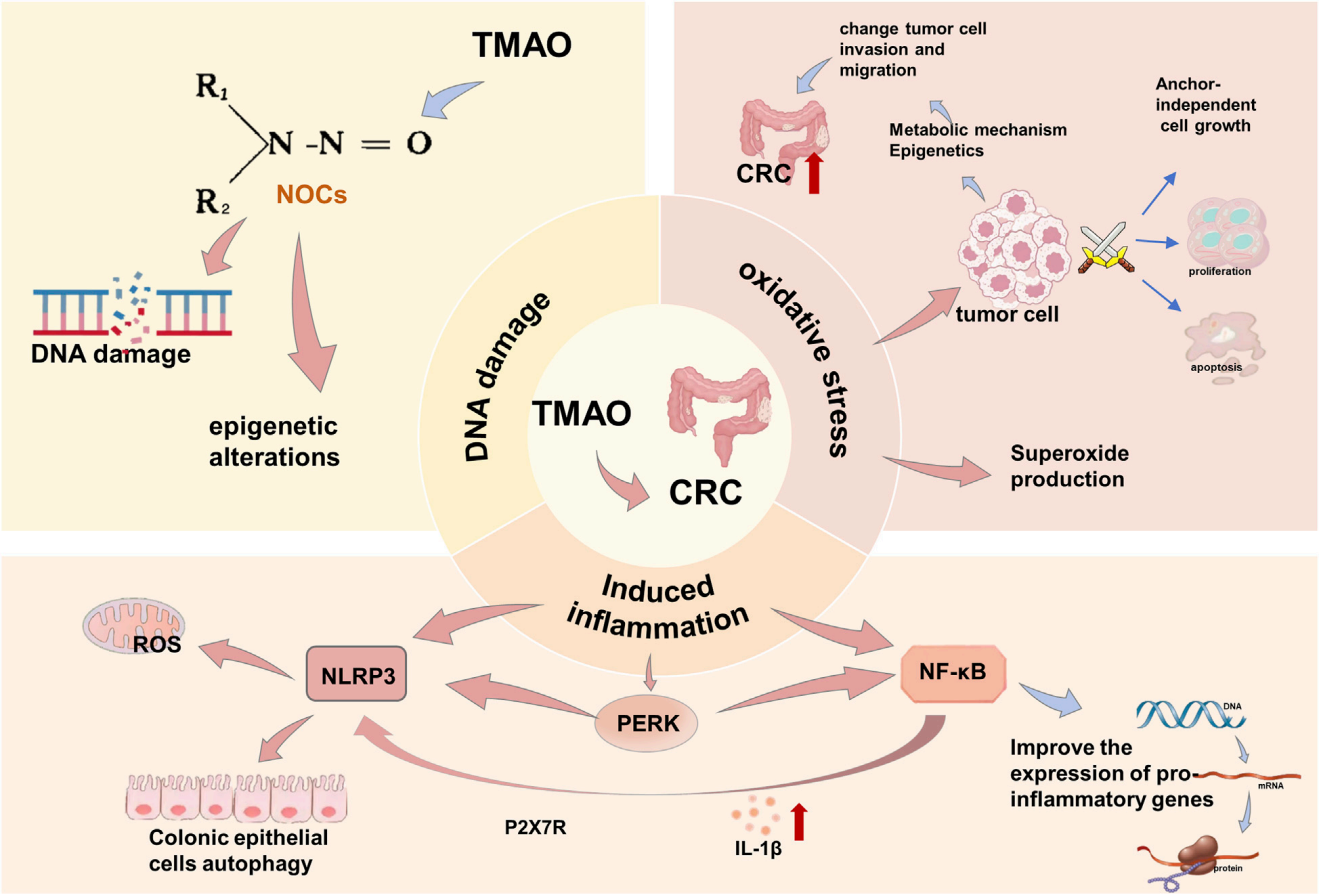
Mechanism of TMAO inducing CRC. TMAO promotes the occurrence and development of CRC by inducing inflammation, oxidative stress, and DNA damage.

Beginning from abnormal crypts, they evolve into precancerous lesions (polyps), eventually progressing to colorectal cancer over an estimated 10–15 years. Currently, most colorectal cancers are supposed to originate from stem cells or stem cell-like cells (Medema, 2013; Nassar and Blanpain, 2016). These cancer stem cells are the result of accumulating genetic and epigenetic changes that inactivate tumor suppressor genes and activate oncogenes. Located at the base of colonic crypts, these cancer stem cells are crucial for tumor initiation and maintenance (Medema, 2013; Nassar and Blanpain, 2016). Studies have found that TMAO may share numerous gene pathways, including immune system, cell cycle, and Wnt signaling pathways, with CRC, indicating a clear link between TMAO concentration and CRC (Xu et al., 2015), however, its specific mechanism requires further investigation. Existing research suggests TMAO influences CRC through inflammation induction, oxidative stress, and DNA damage. One way TMAO promotes colorectal cancer is by inducing inflammation. In a long-term choline-fed mouse experiment, TMAO administration leds to NF-κB-mediated pro-inflammatory responses (Seldin et al., 2016). Enhanced TMAO levels can promote the initiation of the NF-κB pathway and improve the expression of pro-inflammatory genes, including chemokines, adhesion molecules, and inflammatory cytokines. The NLRP3 inflammasome, closely related to CRC, is activated by TMAO-induced endothelial inflammation mediated by mitochondrial reactive oxygen species (ROS) (Chen M. L. et al., 2017). Another study showed that TMAO promotes IBD progression by inhibiting ATG16L1-induced autophagy in colonic epithelial cells, thereby activating the NLRP3 inflammasome (Yue et al., 2017). Moreover, a potential receptor for TMAO may be PERK, which was identified in hepatocytes (Chen S. et al., 2019). Activation of PERK may subsequently lead to the activation of NLRP3 and NF-κB (Chen S. et al., 2019). Specifically, in CRC cells, TMAO has been proved to promote proliferation and potential angiogenesis by upregulating vascular endothelial growth factor A (Yang S. et al., 2022). Therefore, TMAO affects CRC by activating PERK, subsequently activating NLRP3 and NF-κB (Figure 1). In systemic circulation, increased TMAO levels are associated with oxidative stress and induce the production of superoxide, a type of ROS (Li T. et al., 2017). Oxidative stress can render tumor cells insensitive to anti-proliferative signals, apoptosis, and anchorage-independent growth, which alters tumor cell invasion and migration through epigenetic and metabolic mechanisms, further contributing to colorectal cancer development and progression (Zińczuk et al., 2020). Consequently, TMAO also participates in the formation of NOCs, leading to DNA damage and epigenetic changes, indicating a potential role of DNA damage in TMAO’s carcinogenic effects (Oellgaard et al., 2017).

Regarding TCM treatment, there are currently no definitive research conclusions about specific TCM components or compounds treating CRC by affecting TMAO. However, as mentioned above, TMAO influences CRC development through the induction of inflammation, oxidative stress, and DNA damage, with NLRP3 inflammasomes being noteworthy in the context of inflammation induction. Clinical studies have demonstrated that curcumin inhibits NLRP3 inflammasome activation *via* NF-κB-induced P2X7R signalling in macrophages (Kong et al., 2016), and apigenin significantly reduces the mRNA and protein expression of TMAO-induced NLRP3 (Yamagata et al., 2019), which suggests that TCM could potentially treat CRC by impacting the NLRP3 inflammasome, providing a potential pathway.

#### 3.1.2 Non-alcoholic fatty liver disease

**FIGURE 2:**
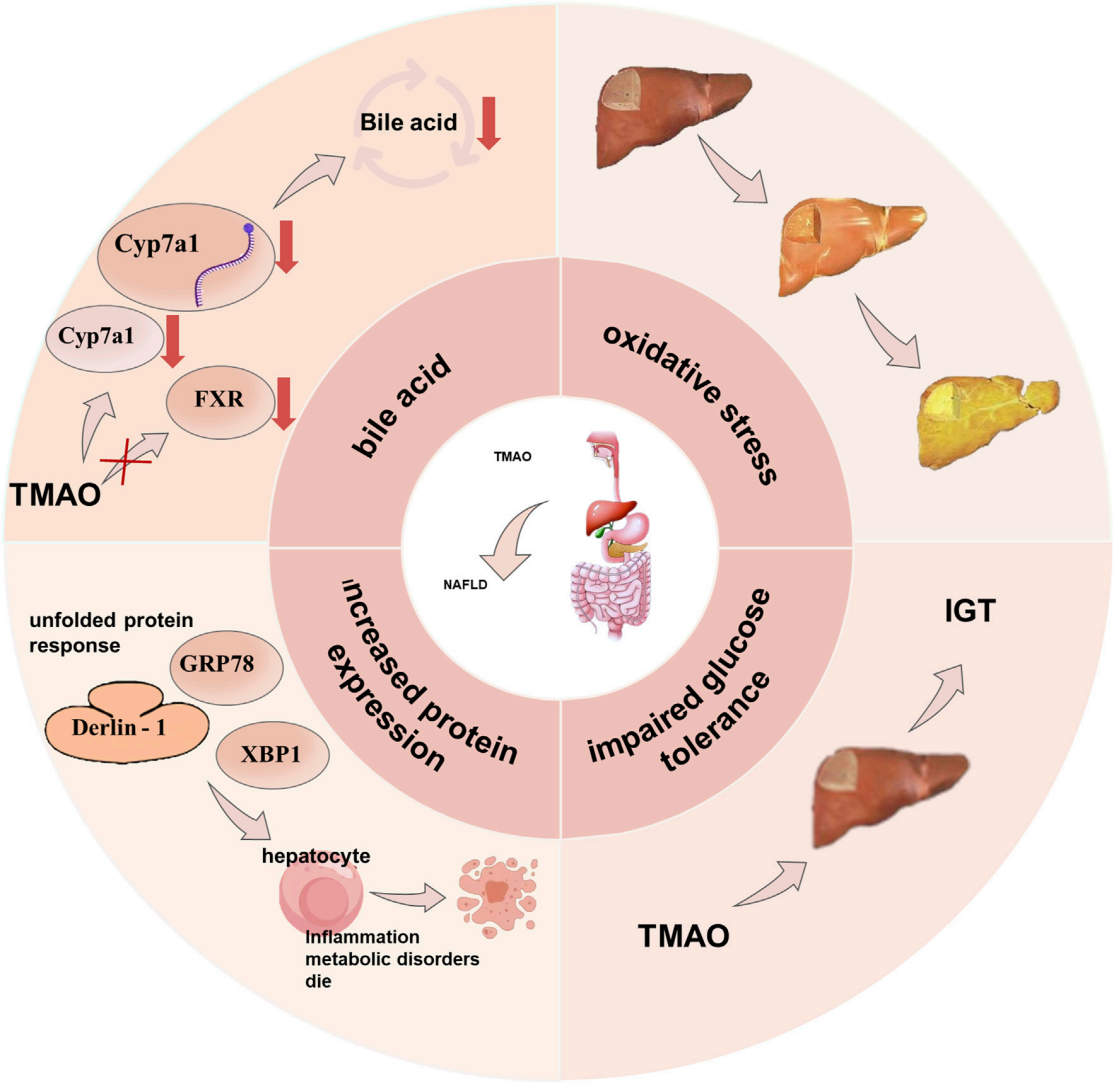
Mechanism of TMAO inducing NAFLD. TMAO causes NAFLD through four pathways: by affecting oxidative stress, by affecting bile acid production, by promoting increased protein expression, and by directly acting on the liver to reduce glucose tolerance.

A key characteristic of NAFLD is hepatic steatosis, mainly driven by obesity, insulin resistance (IR), and adipose tissue (AT) dysfunction. Unhealthy lifestyles and diets high in sugar and fat contribute to obesity and increased liver fat, directly leading to steatosis (Boden, 2006; Powell et al., 2021). IR is characterized by a poor response to insulin whereby glucose uptake is impaired regardless of insulin levels (Boucher et al., 2014). Dysfunctional AT leads to low adiponectin and high leptin levels, causing hepatic insulin resistance and increased lipolysis, further accelarating the development and progression of NAFLD. Studies show that NAFLD patients have higher serum TMAO levels, which correlate positively with steatosis severity (Chen Y. M. et al., 2016; León-Mimila et al., 2021). TMAO is believed to promote NAFLD through four pathways: enhancing oxidative stress (Li X. et al., 2021), impairing glucose tolerance in the liver (Wong et al., 2016; Tang and Hazen, 2017), increasing the expression of proteins related to the unfolded protein response (GRP78, XBP1, Derlin-1) and triggering hepatic lipid metabolism disorders and inflammation (Shi C. et al., 2022), and disrupting bile acid cycling. Specifically, TMAO blocks bile acid-activated farnesoid X receptor (FXR) signaling and reduces key bile acid synthesis enzymes (Cyp7a1, Cyp27a1), limiting bile acid production and increasing fatty liver risk (Koeth et al., 2013; Tan et al., 2019) (Figure 2).

A pivotal aspect of TMAO’s influence on the NAFLD development is bile acid circulation, which provides a new perspective on TCM. Clinical studies have shown that berberine (BBR), a natural plant alkaloid and major pharmacological component in the Chinese herb *Coptis chinensis* Franch, significantly increases secondary or total bile acid content and activates intestinal FXR signalling by accumulating taurocholic acid (TCA) (Tian et al., 2019). Quercetin significantly lowers caecal total bile acid levels (Nie et al., 2019), and *Rubus idaeus* L can promote the expression of bile acid synthesis genes (Matziouridou et al., 2016). Therefore, applying Chinese medicine to regulate bile acid metabolism to treat NAFLD holds substantial promise for the future.

#### 3.1.3 Hypercholesterolemia

**FIGURE 3:**
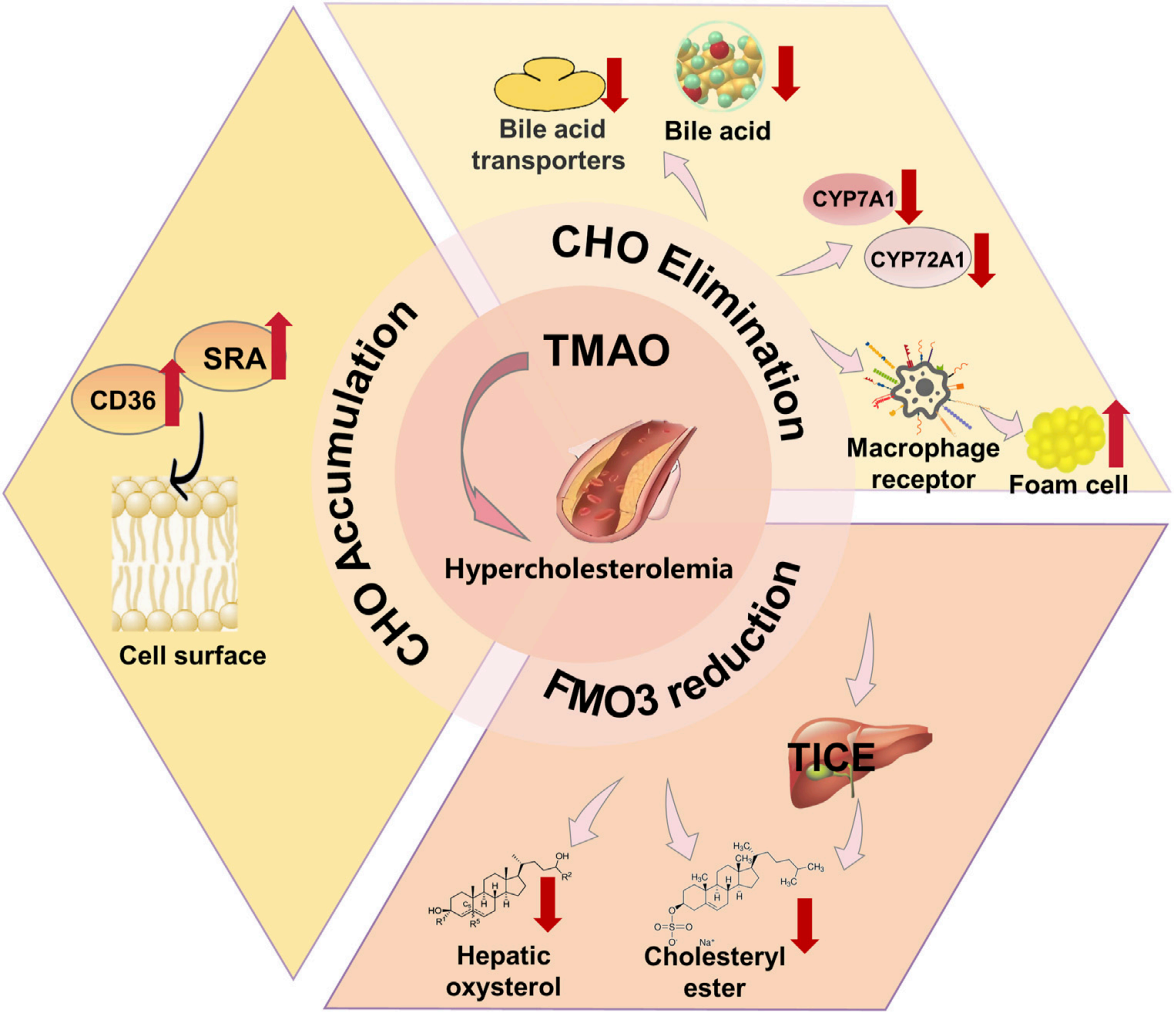
Mechanism of TMAO inducing hypercholesterolemia. TMAO leads to Hypercholesterolemia by affecting cholesterol (CHO) levels and reducing the FMO content.

Hypercholesterolemia is a systemic metabolic disease characterized by abnormal lipid metabolism due to genetic factors, high-fat intake, lack of exercise, *etc.* Relevant studies indicate that TMAO levels are abnormally high in hypercholesterolemia patients, suggesting a close link between TMAO and the pathogenesis of hypercholesterolemia (Dehghan et al., 2020). Previous research shows that TMAO’s impact on hypercholesterolemia is strongly associated with changes in BA metabolism (Duval et al., 2006; Khan et al., 2014; Chen M. L. et al., 2016; Ding et al., 2018). TMAO reduces hepatic bile acid transporter proteins and BA synthesis, effectively decreasing the bile acid pool and promoting a primary pathway for CHO elimination—altering bile acid synthesis, thereby inducing hypercholesterolemia. Studies have shown that TMAO impacts CHO elimination through various mechanisms, leading to hypercholesterolemia. Initially, TMAO promotes foam cell formation through scavenger receptors in macrophages and downregulates main BA synthesizing enzymes cyp7a1 and cyp27a1, reducing intracellular BA levels and affecting hepatic CHO, BA production, and bile secretion, leading to hypercholesterolemia. TMAO also decreases the mRNA expression of Niemann-Pick C1 (NPC1L1) and ATP-binding cassette (ABC) G5/G8, inhibiting intestinal CHO absorption (Koeth et al., 2013). Other studies found that TMAO induces hypercholesterolemia by promoting intracellular CHO accumulation—enhancing macrophage cholesterol accumulation *via* microbiota-dependent pathways involving the increased expression of pro-atherogenic scavenger receptor proteins CD36 and SRA on the cell surface (Wang et al., 2011). Additionally, FMO3 is a negative regulator of macrophage reverse cholesterol transport and a major pathway for TMAO production. FMO3 also contributes to metabolic anomalies by affecting CHO. Experiments that knocking down hepatic FMO3 in LDL receptor-deficient mice demonstrate that FMO3 knockdown can alter bile secretion, intestinal absorption, and constrains hepatic oxysterol and cholesterol ester production in cholesterol-fed mice (Shih et al., 2015; Warrier et al., 2015). FMO3 also impairs cholesterol flux into the TICE pathway, triggering hypercholesterolemia. These studies suggest that FMO3 and TMAO are critical targets for treating hypercholesterolemia (Canyelles et al., 2018), and inhibiting TMAO generation is a crucial treatment method. There are two possible mechanisms to inhibit TMAO production: inhibiting TMA production and reducing FMO3 expression or activity (Figure 3).

Modern research indicates that antibiotics, choline analogues such as DMB, probiotics, plant sterols, and statins have demonstrated effectiveness in treating hypercholesterolemia. These treatments employ different mechanisms, with some targeting the TMA/FMO3/TMAO pathway and others working through alternative routes. Antibiotics, for instance, reduce TMAO levels by inhibiting the microbial production of TMA from dietary precursors. Broad-spectrum antibiotics like ciprofloxacin and metronidazole can almost completely suppress TMAO levels by targeting microbial populations responsible for converting choline, betaine, and L-carnitine into TMA (Wang et al., 2011; Tang et al., 2013). Despite their initial effectiveness, the long-term impact of antibiotics on TMAO levels remains uncertain. Prolonged antibiotic use can lead to the emergence of resistant bacterial strains, increased risks of obesity and cardiovascular events, and intestinal dysbiosis, which disrupts the balance of gut microbiota. Once antibiotics are discontinued, TMAO levels often rise again. Furthermore, antibiotics not only kill harmful bacteria but also affect beneficial ones, further complicating their use as a long-term solution. In contrast, the choline analogue DMB functions as a non-lethal inhibitor of TMA production. It suppresses microbial TMA lyase activities, such as the biological choline TMA lyase CutC (Yang Y. et al., 2022), without interfering with choline uptake into cells. This inhibition reduces TMA formation in cultured microbes, lowers TMA production across various microbial communities in the human body (Wang et al., 2015), and decreases levels of TMA, TMAO, acetate, and propionate *in vivo*. Although promising, DMB’s chemical structure requires further refinement to enhance its effectiveness and safety (Korpela et al., 2016; Heianza et al., 2019). In addition, specific probiotic strains, including *Escherichia coli* ZDY01 and *Lactobacillus* plantarum ZDY04, also demonstrate promising capabilities in reducing circulating TMAO concentrations (Qiu et al., 2018). Despite potential benefits, probiotic applications in disease management remain contentious. Critical research gaps persist in elucidating gut colonization mechanisms and complex interactions between probiotic strains and existing gut microbiota, necessitating comprehensive scientific exploration (Seguro et al., 2021). Unlike the aforementioned treatments, plant sterols and statins do not target the TMA/FMO3/TMAO pathway. Plant sterols, natural molecules derived from plants, offer a non-pharmacological approach to managing abnormal blood lipid levels. While they can help prevent or control hypercholesterolemia, they are not a substitute for pharmaceutical interventions in more severe cases. On the other hand, statins are widely used drugs that inhibit serum cholesterol levels by suppressing 3-hydroxy-3-methylglutaryl-CoA reductase (Sirtori, 2014), thereby reducing the synthesis of mevalonate and cholesterol. Despite their clinical effectiveness, statins are associated with several adverse effects, including myopathy, hyperglycemia, abnormal liver enzymes, and cognitive impairments, which can limit their long-term use.

Traditional Chinese medicine also shows significant efficacy in treating hypercholesterolemia through the TMAO pathway, with superior advantages. *Ligustrum lucidum* W.T.Aiton (LR), also known as kudingcha, is a flavonoid-rich tea-like plant. LR not only prevents the formation of choline-induced TMA and TMAO but also lowers serum TMAO levels by affecting the gut microbiome (Liu S. et al., 2021), thereby modifying the prevalence of specific microbial taxa and modulating their functional characteristics. Concurrently, LR may influence the molecular mechanisms of cholesterol and BA metabolism. As mentioned earlier, TMAO is closely related to changes in BA metabolism. Studies indicate that LR extract reduces liver and serum cholesterol, increases faecal cholesterol and BA excretion, thus effectively alleviating hypercholesterolemia. LR has historically served as a traditional tea in China, characterized by its economic accessibility, ubiquitous availability, simple preparation method, and minimal adverse reactions, akin to several natural products (Wang et al., 2015; Chen M. L. et al., 2016). Therefore, LR may have greater potential in the prevention of hypercholesterolemia. Resveratrol, an anthraquinone terpene compound, is mainly obtained from the TCM *Reynoutria japonica* Houtt. Studies suggest that resveratrol reduces TMA production by reshaping the gut microbiome in mice and modifying the microbial communities in ApoE, thus inhibiting TMAO synthesis and lowering TMAO levels to mitigate TMAO-induced hypercholesterolemia. As previously mentioned, TMAO affects cholesterol metabolism by influencing BA biosynthesis pathways (Koeth et al., 2013), and research by Chen et al. showed that resveratrol significantly decreases ileal FGF15 mRNA and protein levels, leading to increased BA synthesis. This indicates that resveratrol can induce hepatic BA synthesis *via* the gut-liver FXR-FGF15 axis, thereby mitigating TMAO-induced hypercholesterolemia (Chen M. L. et al., 2016).

#### 3.1.4 Diabetes

**FIGURE 4:**
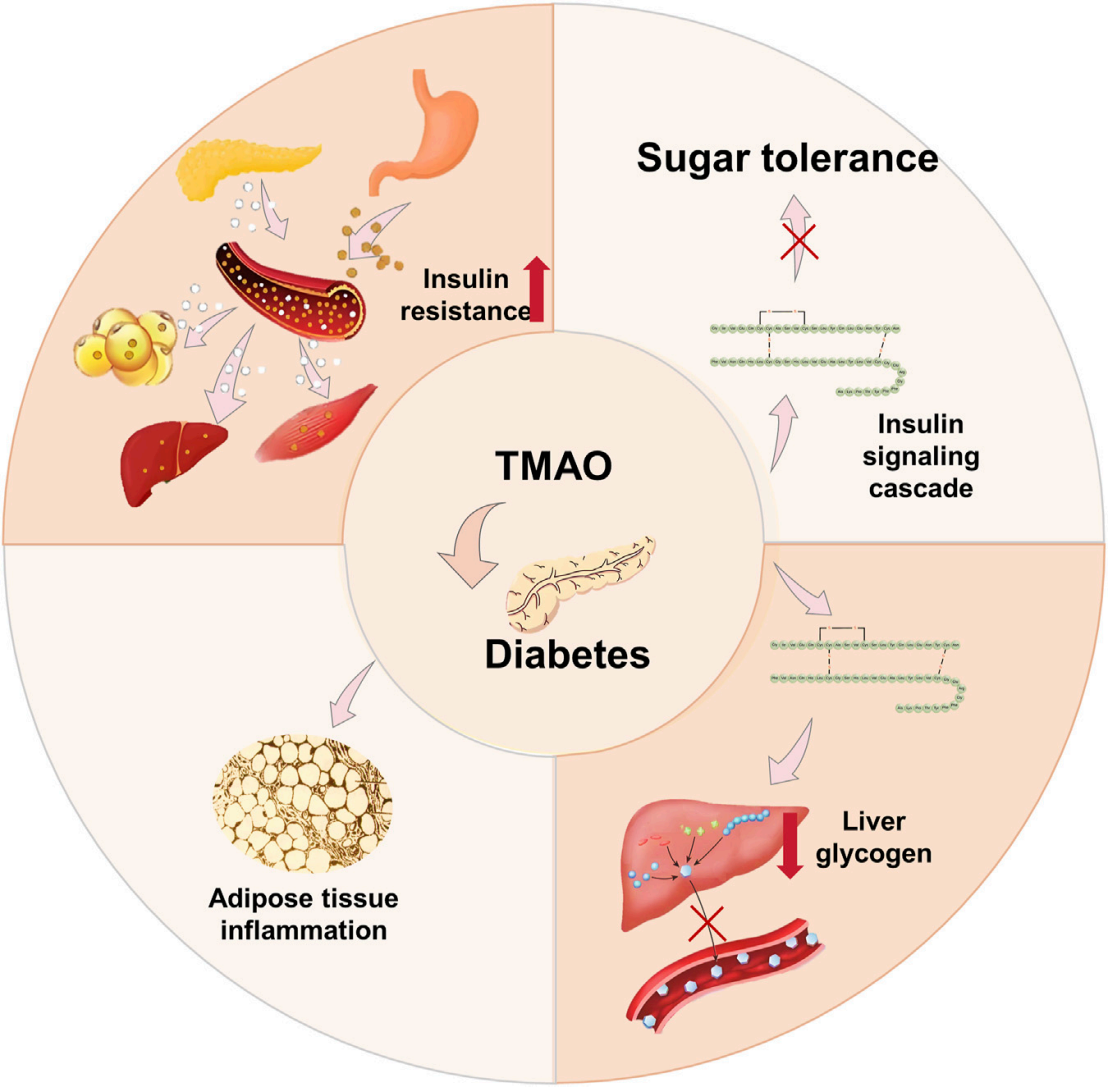
Mechanism of TMAO inducing diabetes. TMAO leads to T2D by increasing insulin resistance (HOMA-IR), inducing adipose tissue inflammation, and aggravating the blockade of the insulin signaling cascade. In addition, TMAO is also associated with genes in the insulin signaling pathway.

Diabetes is a metabolic disorder characterized by persistent hyperglycemia due to impaired insulin secretion or cellular responsiveness. The gut microbiota plays a critical role in type 2 diabetes (T2D) pathogenesis, with T2D patients showing dysbiosis, disrupted intestinal barriers, and abnormal TMAO production and absorption (Wang et al., 2011; Tang et al., 2013). Elevated circulating TMAO is significantly linked to higher T2D risk (Fang et al., 2021b). Mechanistically, TMAO promotes T2D by increasing fasting insulin, HOMA-IR, and glucose intolerance, and inducing adipose tissue inflammation (Gao et al., 2014; Dambrova et al., 2016). It impairs insulin signaling and hepatic glucose metabolism, affecting glycogen synthesis and gluconeogenesis (Kalagi et al., 2022) (Figure 4). TMAO’s production depends on FMO3, and FMO3 polymorphisms may influence T2D risk (Yamazaki and Shimizu, 2013). Nonetheless, the exact mechanism linking circulating TMAO levels to T2D has yet to be fully clarified.

Recent studies suggest that DMB and dietary indoles can prevent and treat diabetes *via* the TMAO pathway. First, DMB, a choline analogue, is found in balsamic vinegar, olive oil, grape seed oil, and red wine. Studies have found that DMB treatment does not affect body weight or lipid abnormalities but significantly reduces plasma TMAO levels and prevents cardiac dysfunction (Chen K. et al., 2017). DMB can mitigate foam cell formation and atherosclerotic plaque development by lowering TMAO levels. However, DMB has been demonstrated solely as an effective non-lethal bactericidal agent, with no proven effect on improving glucose homeostasis and insulin sensitivity. Whereas DMB shows promise in treating cardiovascular disease and T2D, its chemical structure leaves room for improvement. Secondly, dietary indoles can effectively lower TMAO levels and inhibit FMO3 activity, however, their lack of specificity arises from their role as potent 5-HT agonists, with the ability to cross the blood-brain barrier and potentially induce adverse psychological effects (Cashman et al., 1999; Chen J. et al., 2016; Zajdel et al., 2016). Clinically, T2D is commonly treated by insulin injection. Although it shows good efficacy, fear of needles contributes to poor adherence, leading to inadequate blood sugar control and recovery hindrance. Non-invasive alternatives include inhaled or oral insulin; however, challenges exist in using these routes (Zajdel et al., 2016). Clinically, metformin and sulphonylureas are commonly used anti-diabetes drugs. Metformin remains the most widely prescribed antidiabetic agent, particularly for obese and overweight patients. However, metformin exerts no direct effects on β-cells; moreover, in the absence of weight reduction, there is no substantial improvement in muscle insulin sensitivity. Sulphonylureas are secreagogues that treat T2D by triggering the endogenous insulin secretion of pancreatic β-cells. However, sulphonylureas do not have long-term protective effects on β-cell function and may accelerate β-cell failure. Moreover, sulphonylureas, particularly older generations, have a high incidence of causing hypoglycaemia and adverse effects such as weight gain (Gao et al., 2014).

Meanwhile, TCM showed encouraging results in treating diabetes through the TMAO pathway. Flavonoids can lower TMAO levels by inhibiting TMA production, while oolong tea can reduce TMAO levels by reducing FMO3 levels. Studies show an interaction between flavonoids and the gut microbiota, which is directly related to TMAO (Hua et al., 2022). Flavonoids are widely present in a variety of Chinese medicines such as *Styphnolobium japonicum* (L.) Schott, Carthamus tinctorius, *Scutellaria baicalensis* Georgi, Pueraria montana var,*Lonicera japonica* Thunb., *Citrus reticulata* Blanco, *Chrysanthemum × morifolium* (Ramat.) Hemsl., *Epimedium sagittatum* (Siebold and Zucc.) Maxim, *Ginkgo biloba* L., and China’s ten famous traditional green teas, including Lu’an melon seed tea. CutC can break down dietary choline and betaine to generate TMA. Hence, inhibiting CutC activity can suppress TMA production, thereby lowering TMAO levels and achieving anti-diabetic effects. An experiment docking 16 flavonoids from Lu’an melon seed tea with CutC revealed that kaempferol 3-O-rutinoside (Hua et al., 2022), quercetin 3-O-rhamnosidyl galactoside, kaempferol 3-O-rhamnosidyl galactoside, and myricetin 3-O-galactoside can bind with CutC to regulate its activity, suppress TMA production, reduce TMAO levels, and achieve anti-diabetic effects. Additionally, flavonoids, abundant in various plants, fruits, vegetables, and leaves, exhibit a wide range of medicinal properties, such as anticancer, antioxidant, anti-inflammatory, and antiviral activities. They also offer neuroprotective and cardioprotective benefits. Therefore, the advantages of using flavonoids to treat diabetes are more pronounced (Ullah et al., 2020). Oolong tea, a tea variety unique to China, has shown significant efficacy in preventing diabetes. Experiments have indicated that oolong tea extract can lower TMAO formation capacity by remodeling the gut microbiota and downregulating the elevation in FMO3 induced by carnitine (Chen P. Y. et al., 2019). Moreover, FMO3 also reduces lipogenesis and gluconeogenesis through modulating PPARα expression and activity, highlighting the significant efficacy of oolong tea in diabetes prevention (Shih et al., 2015).

### 3.2 Cardiovascular and neurological disorders

Recent research has established that elevated plasma TMAO concentrations demonstrate a significant correlation with an augmented risk of atherosclerotic thrombotic cardiovascular disease (CVD), rendering it a significant pathogenic determinant for cardiovascular, peripheral, and cerebrovascular diseases (Janeiro et al., 2018). TMAO promotes atherosclerosis by upregulating macrophage scavenger receptors (Al-Rubaye et al., 2019), inducing cholesterol accumulation, inflammation, foam cell formation, endothelial dysfunction, and thrombosis (Zhen et al., 2023). Elevated levels of TMAO precursors, such as choline and betaine, are also linked to higher CVD prevalence and poor outcomes. Additionally, TMAO is closely associated with neurological disorders (Parker et al., 2020); it crosses the blood-brain barrier (BBB), and impairs synaptic plasticity and cognitive function by downregulating the mTOR pathway, causing hippocampal neuron loss and synaptic damage (Chen S. et al., 2019).

#### 3.2.1 Atherosclerosis (AS)

**FIGURE 5:**
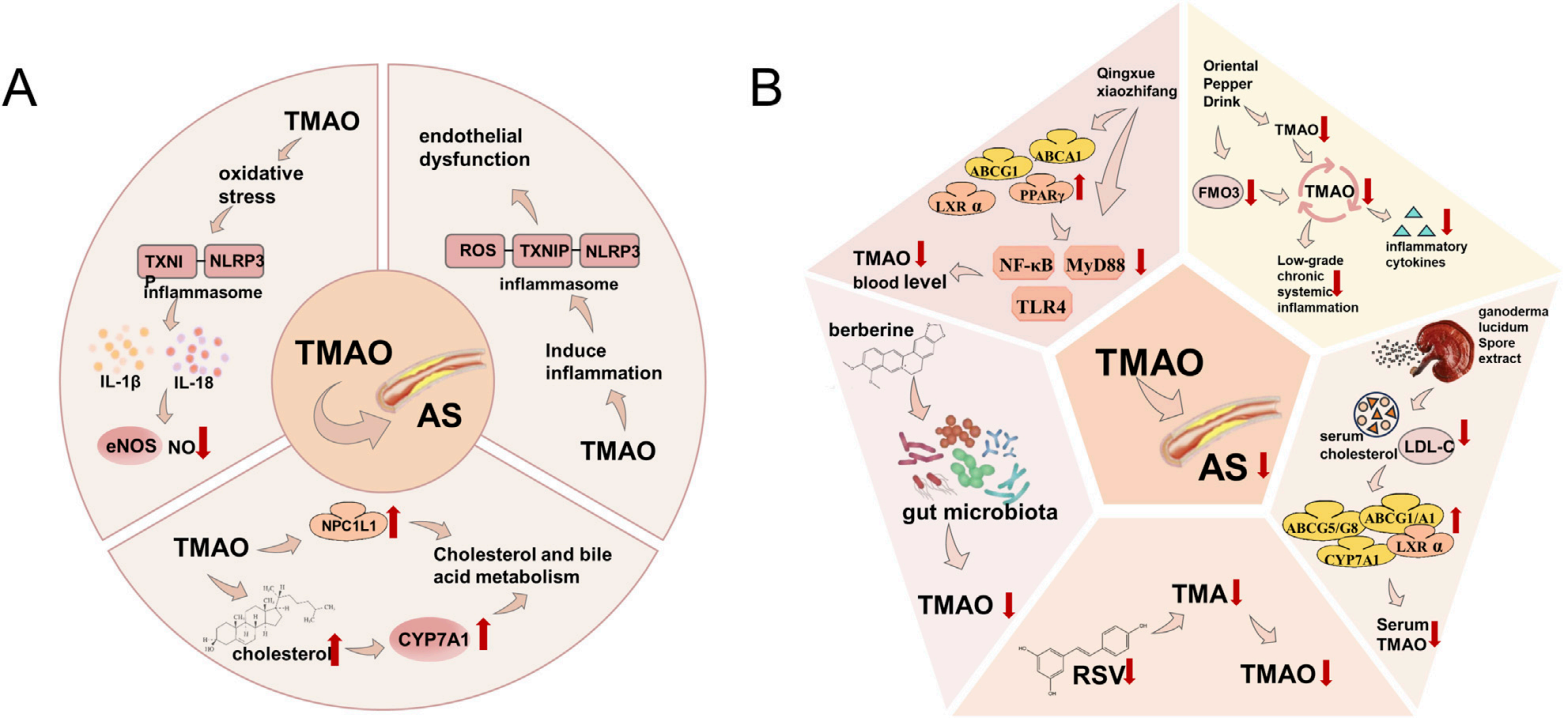
**(A)** Mechanism of TMAO inducing AS. TMAO participates in the occurrence and development of atherosclerosis by inducing oxidative stress, inflammation, and altering the metabolic mechanisms of cholesterol and bile acid in the human body. **(B)** Treatment of AS caused by TMAO by TCM. Traditional Chinese medicine can reduce TMAO by inhibiting inflammation, directly or through inhibiting TMA, regulating gut microbiota, lowering blood cholesterol and LDL-C levels, and reducing FMO3 expression.

In recent years, numerous studies and experiments have shown a close relationship between high levels of TMAO and adverse cardiovascular events caused by atherosclerosis (Komaroff, 2018). TMAO accelerates aortic lesion formation by disrupting cholesterol and bile acid metabolism. In animal models, use of the choline TMA lyase inhibitor iodomethyl choline (IMC) increases bacterial cholesterol metabolite loss and decreases intestinal sterol transport protein Niemann-Pick C1-Like 1 (NPC1L1) expression, reshaping gut microbiota and reducing hepatic cholesterol accumulation, while upregulating CYP7A1 and other bile acid-related genes (Pathak et al., 2020). Mechanistically, TMAO induces oxidative stress and activates the ROS-TXNIP-NLRP3 inflammasome, increasing inflammatory cytokines (IL-1β, IL-18), impairing endothelial nitric oxide synthase (eNOS) and nitric oxide (NO) production, and leading to endothelial dysfunction (Sun et al., 2016). Clinical data show that higher plasma TMAO is associated with increased risk of major adverse cardiovascular events (MACE) and mortality (Guasti et al., 2021). TMAO is now considered both a driver and prognostic marker of atherosclerosis progression to CVD, mainly by affecting lipid metabolism, inflammation, and endothelial function (Wang et al., 2011; Koeth et al., 2013). TMA, produced by gut microbes, is oxidized to TMAO in the liver *via* FMO3(Tang et al., 2013; Shih et al., 2019), triggering chronic inflammation and arterial damage that promote atherosclerotic lesions (Fatkhullina et al., 2018; Komaroff, 2018) (Figure 5A).

In recent years, mechanistically-based small molecule inhibitors targeting the primary bacterial enzyme TMA lyase have been developed, presenting potential as anti-atherosclerotic thrombotic agents (Pathak et al., 2020). TCM can modulate lipid metabolism through altering levels of TMAO. For example, *Zingiber officinale* Roscoe exhibits anti-atherosclerosis effects. Moreover, results from various animal model experiments have indicated that resveratrol (RSV) can mitigate TMAO-induced AS by suppressing TMA formation, with the hepatic FXR-fibroblast growth factor 15 (FGF15) axis serving a critical role in resveratrol-induced bile acid (BA) synthesis (Chen M. L. et al., 2016). Numerous types of Chinese herbs can regulate lipid metabolism in the body (Li et al., 2021c). TMAO is formed through the super-metabolism of dietary substrates containing trimethylamine groups, making its production highly dependent on the composition of the gut microbiome (Falony et al., 2015). Altering dietary habits to reduce the intake of TMAO precursors (such as choline and carnitine) or modifying the composition and function of the gut microbiome can diminish TMAO formation (Iglesias-Carres et al., 2021). A high-choline diet raises TMAO levels and atherosclerosis in animal populations. TMAO may partially mediate the well-documented correlation between red meat intake and cardiovascular disease risk. Therefore, lower blood TMAO levels due to fruit and vegetable intake could explain the cardioprotective effects observed (Sun et al., 2016). Berberine, a bioactive alkaloid derived from traditional Chinese herbal medicines, demonstrates the capacity to suppress TMAO production through modulation of the gut microbiome’s microbial composition. Berberine’s anti-atherosclerotic efficacy potentially stems from its ability to reduce TMAO production, making it an excellent modulator for inhibiting atherosclerotic plaque development (Chen and Wang, 2021). Berberine has emerged as a compound with the potential to lower atherosclerosis risk (Etxeberria et al., 2015). Qing-Xue-Xiao-Zhi Formula (QXXZF) demonstrates significant anti-atherosclerotic effects *in vivo* and *in vitro* by improving lipid metabolism and inhibiting inflammation. It reduces blood TMAO concentration, helping to elucidate its potential mechanisms for atherosclerosis protection. QXXZF can treat atherosclerosis by upregulating the ABCA1/ABCG1-PPARγ/LXR axis and inhibiting the TLR4/MyD88/NF-κB signalling pathway (Li et al., 2021d). *Ganoderma lucidum* (Leyss. ex Fr.) Karst spore extract has hypolipidaemic and anti-atherosclerotic effects on hyperlipidaemic rabbits, lowering blood cholesterol and low-density lipoproteins while reducing arterial plaque area, and upregulating LXR, CYP7A1, and ABCA1/G1 in the liver, intestine, and macrophages (Lai et al., 2020). Ganoderma lucidum spore extract decreases TG, TC, LDL levels, and serum TMAO in heart failure rats induced by high TMAO levels (Liu Y. et al., 2021). The Alisma orientalis Beverage (AOB), a TCM made from various herbal plants, has long been used to treat metabolic syndrome and AS (Liu et al., 2023). In an atherosclerosis model established in male apolipoprotein E-deficient mice fed a high-fat diet (HFD), multiple interventions were applied. Data analysis revealed that after 8 weeks of HFD, AOB-treated mice exhibited significantly reduced inflammatory cytokine expression and AS development. Furthermore, AOB lowered serum TMAO and hepatic FMO3 expression (Figure 5B). Diminishing circulating TMAO levels can mitigate inflammatory cytokine release, thereby attenuating chronic low-grade systemic inflammation and consequently reducing the risk of HFD-induced atherosclerosis. The anti-atherosclerotic effects of AOB are related to changes in the gut microbiome and reduced gut microbiome metabolite TMAO, indicating AOB’s potential therapeutic value in AS treatment (Zhu et al., 2020).

#### 3.2.2 Thrombosis

**FIGURE 6:**
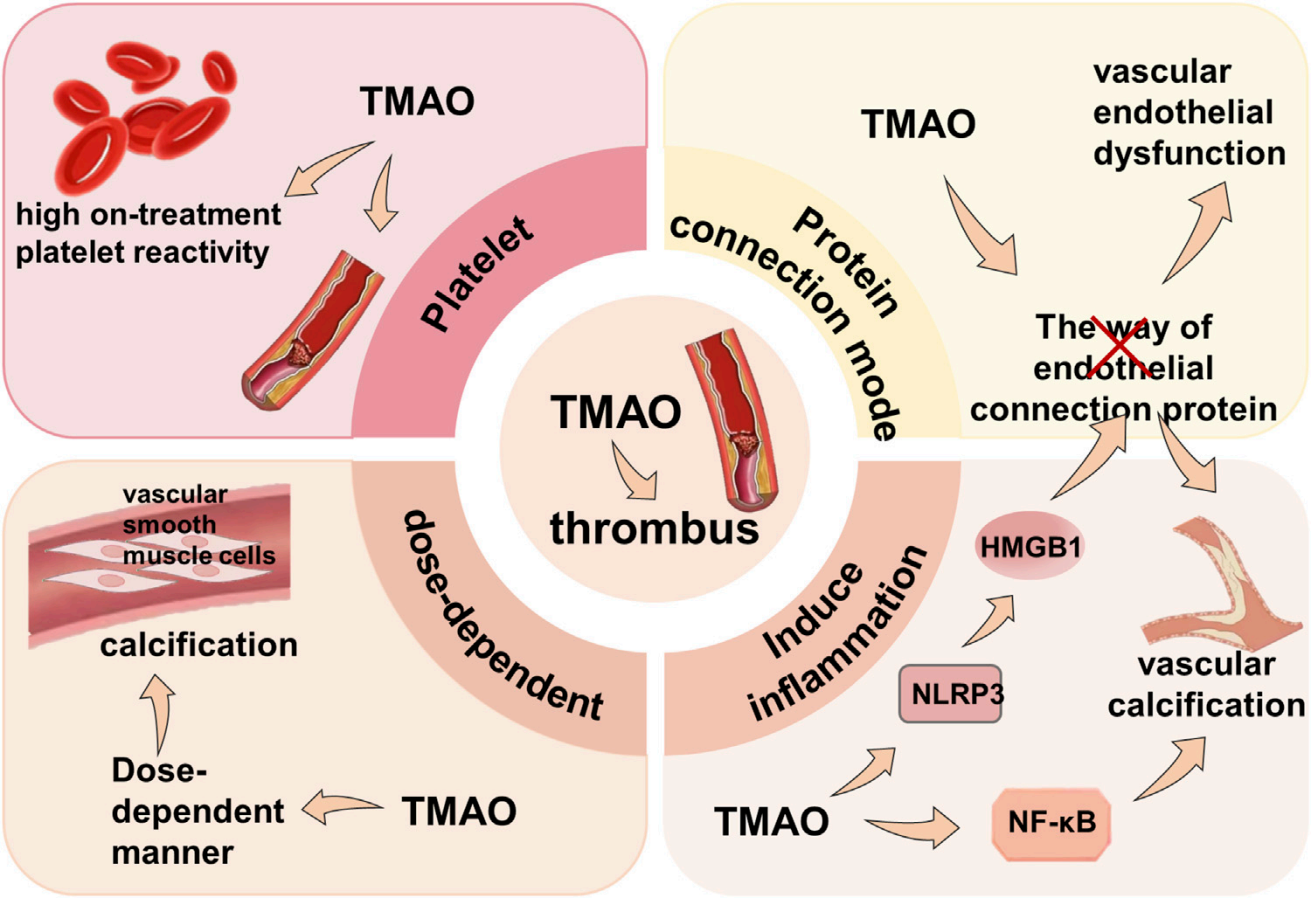
Mechanism of TMAO inducing thrombus. TMAO participates in thrombosis by directly causing high platelet reactivity, disrupting protein connections, activating inflammatory pathways, exacerbating vascular calcification, and promoting vascular calcification in a dose-dependent manner.

Research indicates that adverse cardiovascular outcomes, including arterial thrombosis and mortality (Tang et al., 2013; Li X. S. et al., 2017; Haghikia et al., 2018), are linked to TMAO (Zhu et al., 2018). TMAO promotes platelet hyperactivity, elevates thrombosis risk, and directly regulates thrombotic diseases. After vascular endothelial dysfunction, the exposure of collagen and tissue factor triggers thrombus formation (Furie and Furie, 2008), while vascular calcification (VC) increases vessel rigidity and facilitates thrombosis (Lee et al., 2020). TMAO also induces endothelial dysfunction by disrupting junction proteins, activating the NLRP3 inflammasome to release high-mobility group box 1 protein (HMGB1), and altering endothelial permeability (Singh et al., 2019). Furthermore, TMAO exacerbates VC through dose-dependent vascular smooth muscle cell calcification and activation of the NLRP3 inflammasome and NF-κB signaling (Zhang et al., 2020), thereby further promoting thrombosis (Figure 6).

Given that TMAO can induce thrombosis directly or indirectly through endothelial damage and vascular calcification, lowering TMAO levels and inhibiting factors leading to thrombosis might be effective in treating CVD through drugs or relevant medical interventions. Nonetheless, further research is needed to elucidate specific therapeutic mechanisms. Moreover, HMGB1, a key mediator of TMAO-induced endothelial dysfunction, might serve as a significant target for treating endothelial dysfunction and its related cardiovascular diseases. Cell control experiments and analyses demonstrate that glycyrrhizic acid, a known HMGB1 binder, which reduces HMGB1 expression induced by TMAO, can be used to treat the disruption of cell junction proteins (Singh et al., 2019).

Numerous studies have concluded that TMAO is a non-traditional risk factor for CVD in patients with CKD (Bennett et al., 2013). For these patients with both CVD and CKD, standard clinical interventions for managing CVD do not improve cardiovascular outcomes. Consequently, CVD represents the predominant cause of mortality among patients with CKD (Levey et al., 1998). Experiments have demonstrated that TMAO exhibits acute positive inotropic and lusitropic effects on human and mouse myocardium, significantly increasing intracellular calcium in ventricular cardiomyocytes (Oakley et al., 2020). However, research indicates that chronically elevated contractility and intracellular calcium levels increase cardiac energy consumption, e ultimately leading to heart failure (Böhm et al., 2010).

#### 3.2.3 Cognitive dysfunction

Synaptic plasticity is the activity-dependent change in the strength of neuron connections (Magee and Grienberger, 2020), long believed to be a fundamental component of learning and memory. TMAO reduces synaptic plasticity and induces cognitive dysfunction by promoting endoplasmic reticulum stress and directly binding to activate PERK, which damages synaptic plasticity (Zhang et al., 2007; Chen S. et al., 2019), leading to damage of synaptic plasticity. TMAO also impairs synaptic plasticity through the mammalian target of rapamycin (mTOR)/70-kDa ribosomal protein S6 kinase (p70S6K) pathway, both key regulators of protein translation and synaptic plasticity (Lipton and Sahin, 2014; Zhou et al., 2023). Studies show that elevated TMAO decreases mTOR and p70S6K expression, causes hippocampal neuron loss and synaptic ultrastructural damage, and worsens cognitive function in mice, with higher TMAO leading to more severe deficits (Li et al., 2018). Intervention with L. plantarum alongside memantine reduced Aβ1-42 and Aβ1-40, protected hippocampal neurons, improved synaptic plasticity, and decreased TMAO production, alleviating cognitive impairment in AD mice (Wang et al., 2020). However, clinical trials found that memantine treatment may worsen stuttering and language problems in autistic children (Alaghband-Rad et al., 2013).

In TCM, TMAO mainly alleviates cognitive impairment by improving synaptic plasticity. Liu et al. found through experiments that compared to the TMAO group, synaptic structural damage significantly improved in the AOB group, characterized by regular synaptic morphology, enhanced vesicle distribution within the presynaptic region, and more obvious synaptic clefts. This indicates that AOB mitigates TMAO-induced cognitive impairment by improving synaptic plasticity and regulating synaptic-related proteins (Liu et al., 2023). Furthermore, Jin et al. found that the combination of Danggui Shaoyao San (DSS) and its decoction formula can diminish the prevalence of detrimental gut microbiota (Jin et al., 2023), thereby improving cognitive and learning capacities. Through Nissl staining and Western blot analysis of the integrity of hippocampal neurons and synaptic protein expression, they found that DSS and the decoction formula group showed reduced damage to hippocampal neurons and increased expression levels of synapsin I (P < 0.05) and PSD95 (P < 0.01) proteins. Meanwhile, alpha and beta diversity analyses indicated that the richness and diversity of gut microbiota species in DSS and decoction formula groups were similar to the sham operation group, signifying significant recovery effects (P < 0.05). This suggests that DSS may mitigate cognitive impairment by modulating gut microbiota and increasing the proportion of beneficial bacteria, thereby reducing TMAO production. Additionally, baicalin and berberine have also been shown to lower TMAO levels (Liu et al., 2020). Bazi Bushen capsule alleviates cognitive deficits by inhibiting cellular senescence, secreting SASP factor, and regulating microglial activation and polarization mechanisms, thereby reducing the decline of synaptic function and protecting neurons (Ji et al., 2022).

#### 3.2.4 Alzheimer’s disease (AD)

**FIGURE 7:**
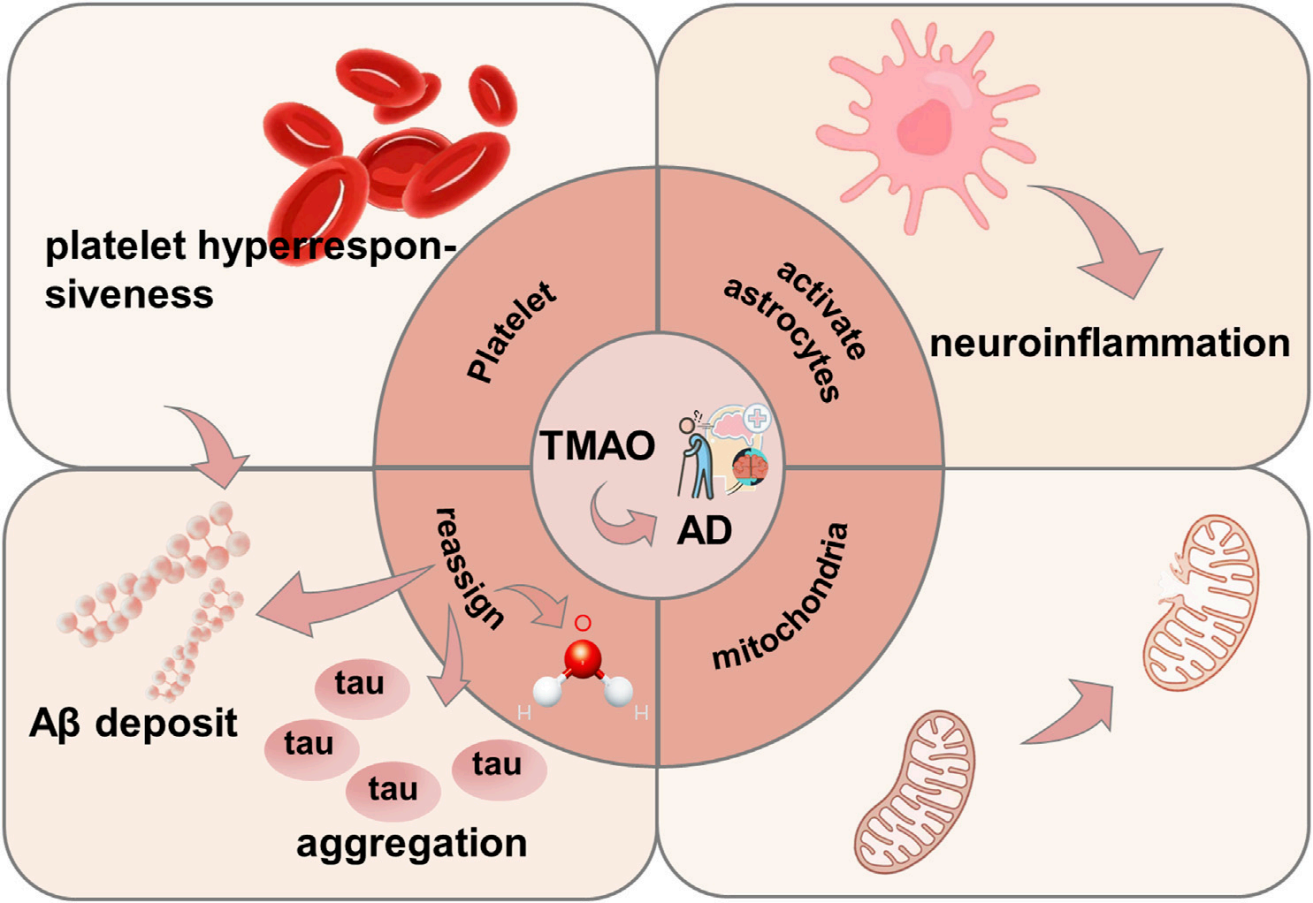
Mechanism of TMAO inducing AD. TMAO promotes AD by directly promoting platelet hyperreactivity, activating astrocytes to induce neuroinflammation, inducing mitochondrial damage, promoting tau protein aggregation and A β deposition.

AD is a progressive neurodegenerative disorder of the central nervous system in the elderly, marked by cognitive impairment and characterized by cerebral amyloid plaques (mainly amyloid β, Aβ) and neurofibrillary tangles (NFT) (Naseri et al., 2019). TMAO promotes Aβ aggregation and stabilizes aggregates by redistributing water and enhancing hydrogen bonding (Kumari et al., 2018). TMAO levels are correlated with hippocampal Aβ plaques, and Aβ accumulation is an early pathological change in AD (Jack et al., 2019). Additionally, TMAO increases platelet reactivity, promoting Aβ release and neuroinflammation (Zhu et al., 2016; Borroni et al., 2002). TMAO also enhances tau aggregation into NFTs by stabilizing hydrogen bonds, reducing the aggregation threshold and lag phase (Levine et al., 2015). Furthermore, TMAO activates astrocytes to secrete pro-inflammatory mediators, leading to neuroinflammation (Heneka et al., 2010; Brunt et al., 2021) Finally, TMAO induces mitochondrial dysfunction, neuronal aging, and mitochondrial damage in the hippocampus, contributing to AD pathology (Swerdlow et al., 2010; 2014; Swerdlow et al., 2017; Li et al., 2018) (Figure 7).

Clinically, common AD medications are acetylcholinesterase inhibitors (AChEIs). However, a trial involving 22,845 AD patients showed that those treated with AChEIs had higher risks of appetite disorders, insomnia, or depression compared to those receiving a placebo (Bittner et al., 2023), indicating that modern clinical drugs come with certain side effects.

Traditional Chinese medicine treats AD primarily by regulating gut microbiota balance to inhibit TMAO production. Guanxinning tablets, an oral compound preparation composed of *Salvia miltiorrhiza* Bunge and *Pueraria montana* var. *lobata* (Willd.) Maesen and S.M.Almeida ex Sanjappa and Predeep [Fabaceae], exhibit potential in mitigating TMAO concentrations and enhancing gut microbiota composition (Zhang et al., 2021). Xanthoceraside (XAN), extracted from the husks of *Xanthoceras sorbifolium* Bunge, was found by Zhou et al. to alleviate gut microbiota imbalance and modulate levels of microbial-derived metabolites to mitigate AD (Zhou et al., 2022). However, the precise mechanism by which XAN treats AD through altering microbial-derived metabolite levels remains unclear, though it may relate to reduced TMAO levels. *Schisandra chinensis* (Turcz.) Baill, isolated from Schisandra chinensis polysaccharides (SCP), and preventative electroacupuncture can regulate gut microbiota by increasing the proportion of beneficial bacteria, thereby suppressing harmful bacteria and reducing TMAO production (He et al., 2021; Fu et al., 2023), preventing the onset of AD. In addition, some traditional Chinese medicine prescriptions can improve the clinical symptoms of AD through other effects. For example, Danggui-Shaoyao-san prescription plays an active and effective role in improving oxidative stress and neuroinflammation in APP/PS1 mice and ultimately improving cognitive deficits, which is conducive to the improvement of AD (Wu Q. et al., 2020). A natural Pterocarpus indicus plant antitoxin, Medicarpin, can alleviate cognitive and memory dysfunction in AD patients by influencing the cholinergic system, neuronal apoptosis and synaptic function (Li D. et al., 2021).

#### 3.2.5 Parkinson’s disease (PD)

PD is a prevalent neurodegenerative disorder affecting the elderly (Khan et al., 2019). Numerous researchers have demonstrated that TMAO serves as an early biomarker for Parkinson’s disease (Chung et al., 2021). TMAO promotes PD progression by inducing neuroinflammation through microglial activation. Elevated serum TMAO not only activates astrocytes in the striatum and hippocampus of the PD models in mice but also promotes M1-type polarization of microglia (Quan et al., 2023), thereby initiating neuroinflammatory cascades. Microglia exhibit phenotypic plasticity, capable of transitioning between the pro-inflammatory M1 phenotype and the anti-inflammatory M2 phenotype (Ji et al., 2018). Qiao and colleagues demonstrated that elevated serum TMAO significantly upregulated mRNA expression of pro-inflammatory M1 microglial markers (CD16, CD32, and iNOS) in the TMAO + MPTP experimental model, suggesting a potential mechanism by which high TMAO levels intensify neuroinflammatory processes in PD through M1 microglial polarization (Qiao et al., 2023). Additionally, TMAO can induce α-synuclein misfolding, affecting neuron cells. Aggregated forms of α-Synuclein in neurons or glial cells are pathological markers of PD (Lücking and Brice, 2000). α-Synuclein is a small (14 kDa), highly conserved presynaptic protein abundant throughout the brain (Maroteaux et al., 1988). Through small-angle X-ray scattering (SAXS) experiments, Uversky et al. analyzed that TMAO induces α-synuclein misfolding (Uversky et al., 2001), leading to PD. Jamal et al. further confirmed through replica exchange molecular dynamics (REMD) simulations that TMAO promotes α-synuclein dense folding (Jamal et al., 2017). Moreover, TMAO can reduce the levels of the neurotransmitter 5-HT, crucial for mood regulation and cognition in the brain (Ni et al., 2021). Quan et al. studied the effect of TMAO on 1-methyl-4-phenyl-1,2,3,6-tetrahydropyridine (MPTP)-induced PD model mice (Quan et al., 2023), determining 5-HT levels and its metabolites in the striatum *via* high-performance liquid chromatography to explore TMAO’s effect on striatal neurotransmitters. Results showed a significant reduction in 5-HT levels in the TMAO + MPTP group compared to the MPTP group, indicating that TMAO diminishes neurotransmitter 5-HT levels, influencing the onset of PD.

Modern medical research suggests L-DOPA is effective for PD treatment. However, prolonged L-DOPA use may lead to motor disorders (Ahlskog and Muenter, 2001), manifesting as involuntary, purposeless, irregular, and repetitive motor phenomena involving limb, axial, and facial musculature. Additionally, antibiotics like metronidazole and ciprofloxacin are frequently used for neurological disorders (Tang et al., 2013), but continuous antibiotic use to reduce TMAO can disrupt intestinal ecology, affecting beneficial gut flora, and TMAO levels can reappear a month or more after stopping antibiotics.

**FIGURE 8:**
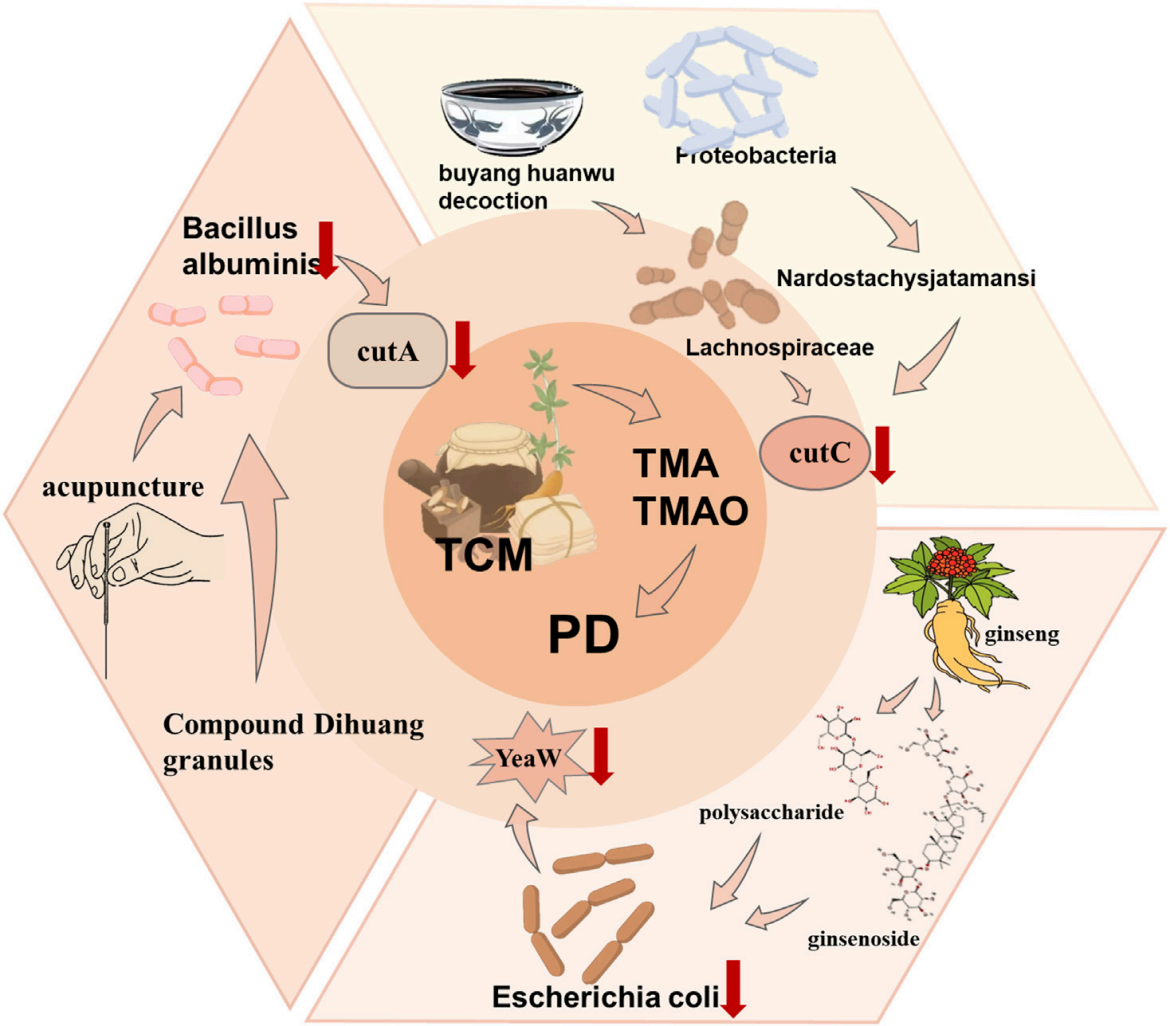
Treatment of AS caused by TMAO by TCM. TCM reduces the TMA level by regulating different intestinal flora and thus reduces the TMAO level to treat PD.

However, TCM offers unique roles in treating PD by reducing the abundance of gut flora that produces TMA, thus indirectly inhibiting TMAO production. TMA is a precursor to TMAO (Galland, 2014), which is oxidized after absorption by intestinal epithelium to form TMAO. Two main TMA biosynthetic mechanisms have been described involving a specialized ethyl radical enzyme (Craciun and Balskus, 2012), The first pathway comprises CutC with its activator CutD, utilizing choline as a substrate, while the second involves a two-component Rieske-type oxygenase/reductase system (CntA/B). Rath et al. established a key gene database for principal TMA synthesis pathways (Rath et al., 2017), encoding CutC and carnitine oxygenase (CntA), to investigate microbial community TMA formation potential. Through 16S rRNA gene sequence analysis, they found that *Clostridium* cluster XIVa and Proteobacteria contain CutC genes, with genes encoding CntA/B found in γ- and β-Proteobacteria. Buyang Huanwu Decoction (BHD) is a renowned TCM formula comprising *Astragalus mongholicus* Bunge, *Angelica sinensis* (Oliv.) Diels, *Paeonia lactiflora* Pall, *Conioselinum anthriscoides ‘Chuanxiong’*, *Carthamus tinctorius* L, *Prunus persica* (L.) Batsch, and Earthworm (Fu et al., 2022). Hu et al. reported that BHD reduces Lachnospiraceae abundance (Hu et al., 2024), a phylogenetically heterogeneous taxon within the Firmicutes phylum of *Clostridium* cluster XIVa (Ruan, 2013). Consequently, BHD reduces Lachnospiraceae abundance, inhibiting TMA production and thus decreasing TMAO levels. Wan et al. found that Astragalus mongholicus not only reduces α-synuclein aggregation in the striatum but also decreases Proteobacteria’s relative abundance in PD models (Wan et al., 2022), indicating Astragalus mongholicus inhibits TMAO production while reducing α-syn aggregation leading to PD. An ancient PD treatment is Compound Dihuang Granule (CDG), composed of seven herbs: *Rehmannia glutinosa* (Gaertn.) Libosch. ex DC., Paeonia lactiflora, *Uncaria rhynchophylla* (Miq.) Miq., Pearl Shell, Salvia miltiorrhiza, *Acorus verus* (L.) Raf., and Scorpion. He et al. found decreased Proteobacteria in PD mice after CDG administration (He Z. Q. et al., 2023), suggesting CDG indirectly reduces TMAO production by lowering Proteobacteria. Polysaccharides and ginsenosides from *Panax quinquefolius* L restore gut microbiota composition (Zhou et al., 2021), reducing *Escherichia coli* abundance. Furthermore, YeaW, a ubiquitous enzyme in *E. coli*, has been proposed as a key enzyme for the third major metabolic pathway of carnitine to TMA conversion (Koeth et al., 2014). Piperine (PIP) stimulates autophagy by inhibiting PI3K/AKT/mTOR activation (Yu et al., 2024), degrading α-synuclein accumulation in PD rat colons and substantia nigra. PIP administration reduces *E. coli* to 6.37%, demonstrating its inhibitory effect on TMAO production. Besides TCM treatment, acupuncture also benefits PD treatment. Acupuncture can improve gut microbiota imbalance; Jang et al. found after acupuncture treatment in PD mice, a reduction in Proteobacteria in the gut, indicating decreased TMAO levels (Jang et al., 2020) (Figure 8).

### 3.3 Urinary system diseases

Plasma levels of TMAO have been found to have a clear association with various human urinary system diseases and serve as a key biomarker for several kidney diseases (Chang et al., 2021). Clinical investigations have consistently demonstrated significantly increased plasma TMAO levels among patients diagnosed with chronic kidney disease (CKD). TMAO suppresses megalin expression in renal tubular epithelial cells through PI3K and ERK signaling pathways, reducing albumin uptake by these cells. Additionally, TMAO induces oxidative stress and activates NLRP3 inflammasomes through the MAPK pathway, thereby impairing renal function and inducing renal fibrosis (Andrikopoulos et al., 2023). TMAO is also a significant factor in diabetic kidney disease (DKD) progression, with elevated plasma TMAO levels leading to NLRP3 inflammasome formation and activation in endothelial cells, which then results in endothelial dysfunction, increased monocyte adhesion, and production of pro-inflammatory cytokines in blood vessels, eventually progressing to vascular oxidative stress and DKD.

#### 3.3.1 Chronic kidney disease (CKD)

**FIGURE 9:**
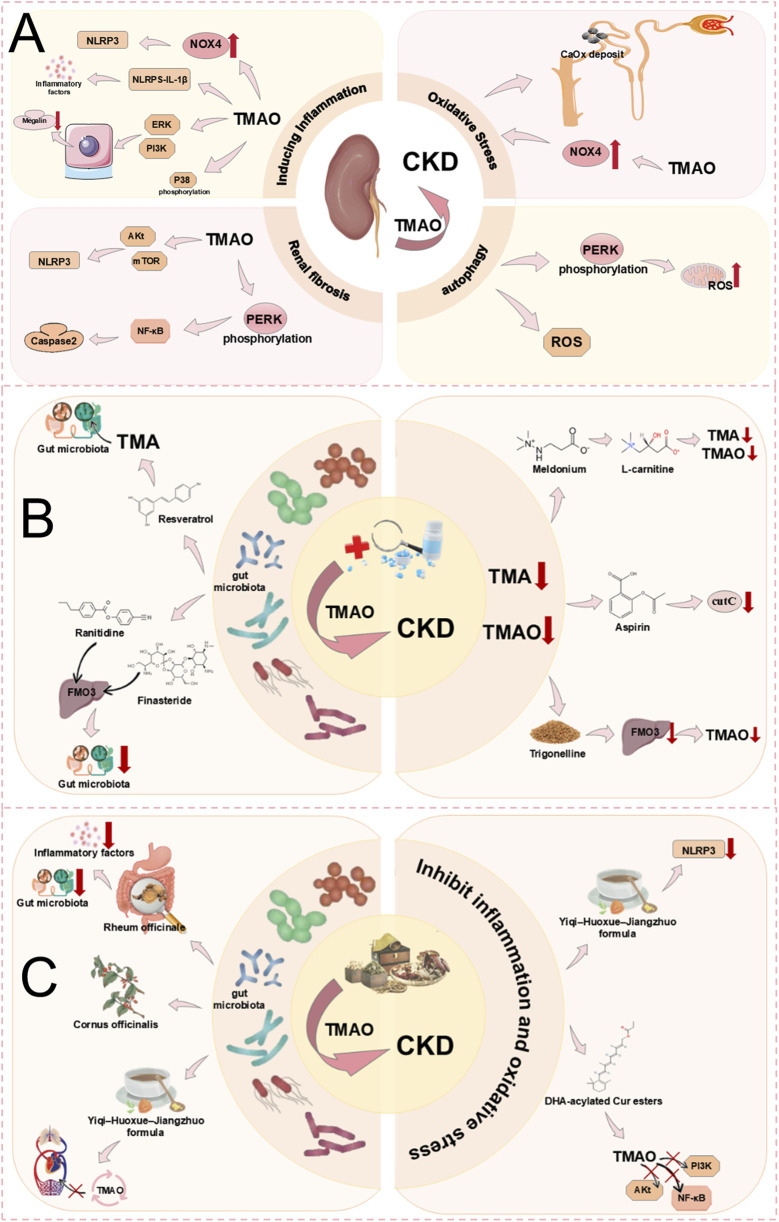
**(A)** Mechanism of TMAO inducing CKD. TMAO promotes renal fibrosis leading to CKD by inducing autophagy, inducing inflammation, and oxidative stress. **(B)** Treatment of CKD caused by TMAO by modern medical drugs. TMAO acts with modern medical drugs to treat CKD through two ways: changing the intestinal microflora and reducing TMA or TMAO precursor levels. **(C)** Treatment of CKD caused by TMAO by TCM. TMAO and TCM treat CKD through two ways: changing intestinal microflora and inhibiting inflammation and oxidative stress.

Metabolomic analysis shows that chronic kidney disease (CKD) patients have higher plasma TMAO concentrations (Prokopienko et al., 2019). In HFD-induced mice, elevated TMAO mediates renal fibrosis and dysfunction *via* oxidative stress and inflammation (Sun et al., 2017). As impaired renal function and fibrosis are key features of CKD (Xu et al., 2017; Wang et al., 2022), TMAO is considered a risk factor. Mechanistically, TMAO inhibits megalin expression *via* PI3K/ERK, reducing albumin uptake and promoting tubular cell dysfunction, which triggers tubulointerstitial inflammation and fibrosis (Kapetanaki et al., 2022). Through promoting p38 phosphorylation in the MAPK pathway, TMAO activates inflammatory pathways and upregulates NOX4 to enhance oxidative stress and activate NLRP3 inflammasomes, resulting in renal inflammation. TMAO also activates the NLRP3-IL-1β axis, promoting inflammatory chemotaxis and cytokine release. Increased oxidative stress and cytokines ultimately lead to tubulointerstitial damage and renal function deterioration, triggering CKD (Lai et al., 2022). Furthermore, TMAO enhances CaOx crystal deposition *via* oxidative stress and autophagy-related cell death, impairs renal function, and promotes kidney stones (Dong et al., 2022). It also triggers fibroblast proliferation and renal fibrosis through PERK/Akt/mTOR and NLRP3/NF-κB signaling, advancing CKD (Kapetanaki et al., 2021) (Figure 9A).

Numerous studies have indicated that TMAO can be a target for the diagnosis and treatment of CKD (Tomlinson and Wheeler, 2017). TMAO is produced by the oxidation of TMA, therefore inhibiting TMA synthesis can effectively reduce TMAO levels. TMAO is related to the abundance of gut microbiota, with several bacterial families, such as Enterobacteriaceae, involved in TMA-TMAO production, making the direct regulation of gut microbiota one method to modulate TMAO levels (Zixin et al., 2022). Medications like ranitidine and finasteride, as FMO substrates, competitively inhibit TMA binding, consequently diminishing TMAO generation and potentially mitigating CKD progression. Additionally, ranitidine and finasteride can significantly decrease Enterobacteriaceae, altering the gut microbiota and offering potential renal protective mechanisms (Zixin et al., 2022). Meldonium can reduce the gut microbiota-dependent production of TMA/TMAO from L-carnitine, thereby lowering TMAO levels and potentially delaying CKD, thereby lowering TMAO levels and potentially delaying CKD (Kuka et al., 2014). However, prolonged use of Meldonium may lead to adverse effects such as hypoxia, dizziness, and a lack of blood supply (Benedetto et al., 2008). A nutritional supplement, RSV, lowers TMAO levels by reducing the gut microbial production of TMA, thus delaying CKD (Song et al., 2020). Aspirin reduces TMAO levels through inhibition of microbial TMA lyase activity, thus delaying CKD progression (Zixin et al., 2022). Trigonelline, an alkaloid extracted from fenugreek seeds, can reduce hepatic FMO3 activity, inhibit TMA oxidation, and decrease TMAO production, thereby slowing the progression of CKD (Yong et al., 2023) (Figure 9B). However, inhibiting FMO3 can also bring about side effects such as liver inflammation and fish odour syndrome (trimethylaminuria) (Wang et al., 2015). Despite significant advances in modern medicine for treating CKD, the side effects associated with the use of modern medications cannot be overlooked.

Additionally, clinical studies have shown that TCM has a significant effect on the treatment of CKD. Curcumin diester acylated with DHA can markedly reduce TMAO levels. DHA-acylated Curcumin diester potently suppresses inflammation, apoptosis, and oxidative stress by interrupting the TMAO-mediated PI3K/Akt/NF-κB signaling cascade (Shi H. H. et al., 2022). This can slow the progression of CKD. The Yi Qi Huo Xue Jiang Zhuo formula (YHJF) consists of five traditional Chinese herbs: Astragalus mongholicus, Angelica sinensis, *Rheum officinale* Baill, Salvia miltiorrhiza, and *Scleromitrion diffusum* (Willd.) R.J.Wang. YHJF demonstrates anti-inflammatory properties by suppressing NLRP3 inflammasome activation, thereby preventing TMAO level escalation in 5/6 nephrectomised mice. YHJF can also alter the gut microbiota and reverse gut permeability, preventing increased transport of TMAO into the circulation and retarding the progression of CKD to some extent (Liu et al., 2022). Rheum officinale enema can also reduce TMAO and TMA levels in the serum of 5/6Nx CKD rats by decreasing certain TMAO-related bacteria, inhibit the expression of inflammatory markers (interleukin-6, tumour necrosis factor-α, and interferon-γ), alleviate renal interstitial fibrosis, and slow the progression of CKD (Ji et al., 2021). Additionally, *Cornus officinalis* Siebold and Zucc. demonstrates potential CKD prevention through comprehensive modulation of gut microbiota and targeted regulation of uraemic toxins (including TMAO) (Du et al., 2022) (Figure 9C).

#### 3.3.2 Diabetic kidney disease (DKD)

**FIGURE 10:**
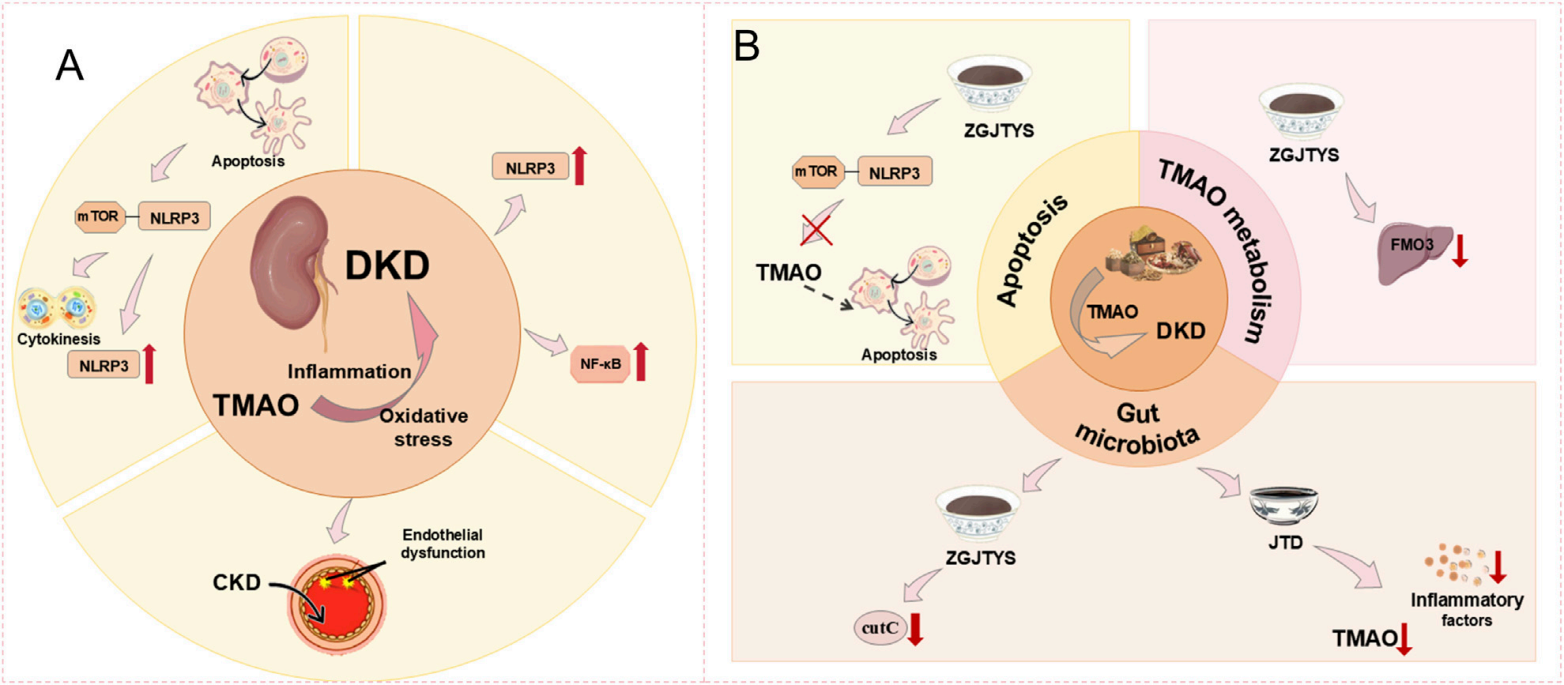
**(A)** Mechanism of TMAO inducing DKD. TMAO leads to DKD by inducing inflammation and promoting oxidative stress. **(B)** Treatment of DKD caused by TMAO by TCM. TCM treats DKD by acting with TMAO by regulating intestinal microbes, promoting cell apoptosis, and affecting TMAO metabolism.

According to the definition, DKD pathogenesis is characterized by compromised renal function, manifesting as either decreased glomerular filtration rate or elevated urinary albumin excretion, or both (Gheith et al., 2016). The Framingham Heart Study suggests that TMAO might be a surrogate marker for GFR. Experiments by Xu and others have also demonstrated that a lower GFR leads to higher TMAO levels (Xu et al., 2017). Therefore, TMAO is highly likely to be a significant risk factor in the onset of DKD. Gut-derived TMAO induces apoptosis by increasing intracellular mROS levels and further promoting NLRP3 assembly activation, with the underlying mechanism being the regulation of the intracellular mROS-NLRP3 axis to activate cytokinesis and release inflammatory factors, thus promoting DKD (Yi et al., 2023). In rats with CKD, augmented TMAO concentrations precipitate vascular oxidative stress and inflammatory cascades, culminating in endothelial impairment. Inflammatory processes and microvascular endothelial compromise constitute pivotal pathogenetic mechanisms in diabetic nephropathy. Therefore, increased TMAO levels can lead CKD patients to develop DKD further. Additionally, TMAO can promote the development of DKD by inducing inflammation (NF-κB, NLRP3, TNF-α, IL-1β, IL-6), oxidative stress, and fibrosis in the renal system (Fang et al., 2021b). The promotion of renal-related inflammatory mechanisms occurs through TMAO activating NLRP3 inflammasomes and NF-κB signal transduction, with NF-κB playing a role in the DKD process by promoting vascular inflammation and oxidative stress, becoming a potential pathogenic mechanism in DKD (Huang et al., 2023) (Figure 10A).

Experiments have demonstrated that elevated serum TMAO levels are positively correlated with the risk of DKD in patients, with TMAO potentially being a biological marker for DKD (Huang et al., 2023). Therefore, targeting TMAO could be a way to treat DKD. However, clear research results regarding the interaction of current modern medical drugs with TMAO to treat DKD are not yet available. Recent studies, however, have shown that high levels of circulating TMAO may exacerbate DKD, suggesting that choline-TMA lyase inhibitors (such as DMB, IMC) might have potential in improving DKD (Fang et al., 2021a). More detailed mechanisms are yet to be widely researched. Encouragingly, there is currently evidence that TCM has a positive effect on the treatment of DKD. The Zuogui-Jiangtang-Yishen Decoction (ZGJTYS) is composed of nine Chinese herbs, including Astragalus mongholicus, Salvia miltiorrhiza, *Dioscorea polystachya* Turcz., Coptis chinensis, *Achyranthes bidentata* Blume, *Leonurus japonicus* Houtt, Rehmannia glutinosa, Cornus officinalis, and *Zea mays* L. It operates from three aspects to provide treatment effects. ZGJTYS may impact the expression of CutC by regulating gut microbiota and inhibit liver FMO3 levels to reduce TMAO. ZGJTYS demonstrates the capacity to directly attenuate TMAO concentrations in both plasma and renal tissue. Moreover, ZGJTYS mitigates diabetic kidney disease progression by suppressing TMAO-mediated apoptotic mechanisms through modulation of the mROS-NLRP3 inflammatory pathway (Yi et al., 2023). The Jiangtang Decoction (JTD) consists of five herbs, including *Euphorbia pekinensis* Rupr., Salvia miltiorrhiza, Astragalus mongholicus, leeches, and *Cistanche deserticola* Ma. JTD reduces TMAO levels and shows alleviating effects on inflammatory factors (such as NLRP3, IL-6, and IL-17A), effectively improving the progression of DKD. JTD regulates the composition of gut microbiota, thereby reducing the potential role of gut microbiota-mediated uraemic toxins (including TMAO) and inflammation in promoting the development of DKD. Although experimental results demonstrate a significant association between JTD and gut microbiota, the underlying mechanisms require further research (Hong et al., 2023).

In the study of kidney diseases, TMAO often induces kidney dysfunction and a decrease in glomerular filtration rate through pathways like activating autophagy, inducing inflammation, promoting renal interstitial fibrosis, inducing apoptosis, and oxidative stress. This mediates the onset and progression of renal diseases such as CKD and DKD. Notably, while modern medical drugs are often accompanied by some adverse reactions, the application of TCM shows certain advantages in treating kidney diseases. Therefore, the extensive use of TCM could potentially become an effective means of treating kidney-related diseases. However, up to now, the deeper pathogenic mechanisms between TMAO, CKD, and DKD, along with the therapeutic mechanisms of TCM interacting with TMAO, have not been fully elucidated. Therefore, further investigation into the development of TMAO and kidney diseases is required (Figure 10B).

## 4 Conclusion and outlook

TMAO, a key metabolite of gut microbiota, has been identified by numerous studies as a common pathological basis for multisystem chronic diseases. Its production relies on the action of CutC in gut microbiota and the oxidation by hepatic FMO3 enzyme. In digestive diseases, TMAO promotes CRC progression through inflammatory responses and oxidative stress. It activates PERK to further stimulate NLRP3 and NF-κB pathways, driving CRC development *via* ROS-mediated oxidative stress. Alternatively, TMAO may increase NAFLD risk by inducing oxidative stress, impairing glucose tolerance, or reducing bile acid synthesis. In cardiovascular diseases, elevated plasma TMAO upregulates scavenger receptors on macrophages to promote cholesterol deposition. Concurrently, TMAO activates the ROS-TXNIP-NLRP3 pathway, causing endothelial inflammatory dysfunction and accelerating atherosclerotic plaque formation. It also disrupts tight junction proteins through inflammation induction, exacerbates VC *via* NLRP3 inflammasome and NF-κB signaling activation, and promotes thrombosis. In neurodegenerative diseases, TMAO penetrates the BBB, impairing synaptic plasticity through PERK activation or mTOR pathway inhibition (Zhou et al., 2023), while promoting Aβ and tau protein aggregation in Alzheimer’s and Parkinson’s disease progression. As a key biomarker in urinary system diseases, TMAO suppresses Megalin expression *via* PI3K and ERK signaling, activates the MAPK-NLRP3 pathway to induce renal oxidative stress and inflammation, and ultimately leads to renal fibrosis.

In TMAO-targeted therapies, TCM demonstrates multi-target intervention capabilities. BBR from Coptis chinensis inhibits TMA oxidation by activating intestinal FXR signaling. Flavonoids competitively block CutC enzyme activity, reducing TMA production at its source. Guanxinning Tablets modulate gut microbiota, while SCP decrease TMA precursor generation. For metabolic and inflammatory regulation, ZGJTYS suppresses TMAO-induced renal apoptosis by inhibiting the mROS-NLRP3 axis. Curcumin inhibits NLRP3 inflammasome activation *via* P2X7R signaling, and apigenin reduces NLRP3 expression to improve cholesterol metabolism disorders. Synergistic effects of TCM formulations are particularly notable: JTD alleviates inflammatory cytokines, while AOB mitigates TMAO-induced cognitive impairment by enhancing synaptic plasticity and regulating synaptic proteins (Zhu et al., 2020; Liu et al., 2023).

In modern medical approaches, prolonged antibiotic use and Meldonium carry significant side effects. While FMO3 inhibitors reduce TMAO levels, they risk adverse effects like hepatic inflammation, and individuals with FMO3 gene defects may develop trimethylaminuria (fish odour syndrome). In contrast, TCM achieves therapeutic outcomes with reduced toxicity risks. For instance, berberine lowers serum TMAO while maintaining gut microbiota homeostasis, and Guanxinning Tablets provide cardiovascular protection comparable to statins without typical statin-related side effects.

However, current research on TCM interventions for TMAO-related diseases faces notable limitations. The mechanism of XAN in treating AD *via* microbiota metabolite regulation remains unclear, as does ZGJTYS’s anti-renal apoptosis mechanism. Additionally, studies exploring TCM-TMAO interactions in systemic disease management remain scarce, highlighting the need for broader experimental and clinical validation.

Existing literature shows promising results but requires more comprehensive experimental and clinical verification. Future research should focus on elucidating TMAO’s pathogenic mechanisms and exploring TCM’s therapeutic potential. Clinically, personalized treatment strategies could be developed by integrating modern medicine with TCM or combining herbal and synthetic drugs to enhance therapeutic efficacy and improve patient outcomes.

The effects of various TCM therapies on different diseases induced by TMAO, including their therapeutic targets and outcomes, are summarized and presented in the following table.

**Table:** 

Disease type	TCM/Components	Target/Mechanism of action	Therapeutic outcome
Colorectal Cancer (CRC)	Curcumin	Inhibit NF-κB-P2X7R signaling and reduce NLRP3 inflammasome activation	Alleviate inflammation and oxidative stress, and improve cell damage
	Apigenin	Reduce the expression of NLRP3 mRNA and protein induced by TMAO	Inhibit inflammatory response and slow down CRC progression
Non-Alcoholic Fatty Liver Disease (NAFLD)	Berberine (BBR)	Activate intestinal FXR signaling and regulate bile acid metabolism	Reduce liver fat accumulation and improve lipid metabolism
	Quercetin	Reduce total cecal bile acid levels	Improve bile acid circulation and alleviate NAFLD
Atherosclerosis (AS)	Qingxue Xiaozhi Formula (QXXZF)	Upregulate ABCA1/ABCG1-PPARγ/LXR axis and inhibit TLR4/MyD88/NF-κB pathway	Reduce TMAO levels and plaque formation
	Ganoderma lucidum spore extract	Upregulate hepatic LXR, CYP7A1, and ABCA1/G1 expression	Reduce cholesterol and LDL, and alleviate atherosclerosis
Thrombosis	Glycyrrhizic Acid	Inhibit TMAO-induced HMGB1 expression and protect endothelial cell junctions	Reduce thrombosis risk and improve endothelial function
Cognitive Impairment	Alisma orientalis Beverage (AOB)	Regulate gut microbiota and reduce TMAO production	Improve synaptic plasticity and alleviate cognitive impairment
	Danggui Shaoyao San (DSS)	Regulate gut microbiota and increase the proportion of beneficial bacteria	Reduce TMAO and protect hippocampal neurons
Alzheimer’s Disease (AD)	Guanximing Tablets	Regulate gut microbiota and reduce TMAO levels	Reduce Aβ deposition and improve cognitive function
	Schisandra chinensis polysaccharides (SCP)	Increase beneficial bacteria and inhibit harmful bacteria	Reduce TMAO and delay the progression of AD
Parkinson’s Disease (PD)	Buyang Huanwu Decoction (BHD)	Reduce Firmicutes (e.g., Lachnospiraceae)	Inhibit TMA production and improve motor dysfunction
	Compound Rehmannia Granules (CDG)	Reduce the abundance of Proteobacteria	Reduce α-synuclein aggregation
Chronic Kidney Disease (CKD)	Yiqi Huoxue Jiangzhuo Formula (YHJF)	Inhibit NLRP3 inflammasome and regulate intestinal permeability	Reduce TMAO entry into the blood and delay renal fibrosis
	Rhubarb Enema	Reduce TMAO-related microbiota and inhibit inflammatory factors (IL-6, TNF-α)	Improve renal function and reduce uremic toxins
Diabetic Kidney Disease (DKD)	Zuogui Jiangtang Yishen Decoction (ZGJTYS)	Inhibit mROS-NLRP3 axis and regulate gut microbiota and hepatic FMO3	Reduce renal cell apoptosis and improve glucose metabolism
Hypercholesterolemia	Kudingcha (Ligustrum robustum)	Regulate cholesterol and bile acid metabolism and inhibit intestinal TMA production	Reduce serum TMAO and cholesterol levels
	Resveratrol	Promote bile acid synthesis through the gut-liver FXR-FGF15 axis	Improve cholesterol excretion
Type 2 Diabetes (T2D)	Flavonoids (e.g., Quercetin, Kaempferol)	Competitive inhibition of CutC enzyme activity and reduce TMA production	Reduce TMAO and improve insulin resistance
	Oolong Tea Extract	Downregulate FMO3 expression and regulate gut microbiota	Reduce TMAO production and improve glucose metabolism

## Glossary

TMAO: Trimethylamine N-oxide
TCM: Traditional Chinese Medicine
FMO3: flavin-containing monooxygenase 3
TMA: trimethylamine
CutC: choline TMA lyase
DMA: dimethylamine
OCT2: Organic cation transporter 2
TMADH: trimethylamine dehydrogenase
ACE-I: angiotensin-converting enzyme inhibitor
CRC: colorectal cancer
NAFLD: non-alcoholic fatty liver disease
PERK: protein kinase R-like endoplasmic reticulum kinase
IR: insulin resistance
AT: adipose tissue
FXR: farnesoid X receptor
BBR: berberine
TCA: taurocholic acid
CVD: cardiovascular disease
AS: Atherosclerosis
NPC1L1: Niemann-Pick C1-Like one
IL: interleukin
eNOS: endothelial nitric oxide synthase
NO: nitric oxide
NAC: N-acetylcysteine
MACE: major adverse cardiovascular events
RSV: resveratrol
FGF15: FXR-fibroblast growth factor 15
BA: bile acid
QXXZF: Qing-Xue-Xiao-Zhi Formula
AOB: Alisma orientalis Beverage
HFD: high-fat diet
VC: vascular calcification
HMGB1: high-mobility group box 1 protein
BBB: blood-brain barrier
mTOR: mammalian target of rapamycin
p70S6K: 70-kDa ribosomal protein S6 kinase
4E-BP1: 4E-binding protein 1
LTP: long-term potentiation
AD: Alzheimer’s Disease
NFT: neurofibrillary tangles
Aβ: amyloid β
ROS: reactive oxygen species
AChEIs: acetylcholinesterase inhibitors
XAN: Xanthoceraside
SCP: Schisandra chinensis polysaccharides
PD: Parkinson’s Disease
SAXS: small-angle X-ray scattering
REMD: replica exchange molecular dynamics
BHD: Buyang Huanwu Decoction
CDG: Dihuang Granule
PIP: Piperine
CKD: chronic kidney disease
DKD: diabetic kidney disease
YHJF: Yi Qi Huo Xue Jiang Zhuo formula
ZGJTYS: Zuogui-Jiangtang-Yishen Decoction
JTD: Jiangtang Decoction
RCT: reverse cholesterol transport
NPC1L1: Niemann-Pick C1
ABC: ATP-binding cassette
CHO: cholesterol
LR: Ligustrum robustum
T2D: type 2 diabetes
DMB: 3,3-dimethyl-1-butanol
